# *flt1* inactivation promotes zebrafish cardiac regeneration by enhancing endothelial activity and limiting the fibrotic response

**DOI:** 10.1242/dev.203028

**Published:** 2024-11-29

**Authors:** Zhen-Yu Wang, Armaan Mehra, Qian-Chen Wang, Savita Gupta, Agatha Ribeiro da Silva, Thomas Juan, Stefan Günther, Mario Looso, Jan Detleffsen, Didier Y. R. Stainier, Rubén Marín-Juez

**Affiliations:** ^1^ Department of Developmental Genetics, Max Planck Institute for Heart and Lung Research, Ludwigstrasse 43, 61231 Bad Nauheim, Germany; ^2^ German Centre for Cardiovascular Research (DZHK) Partner Site Rhine-Main, Max Planck Institute for Heart and Lung Research, 61231 Bad Nauheim, Germany; ^3^ Cardio-Pulmonary Institute (CPI), 61231 Bad Nauheim, Germany; ^4^ Bioinformatics and Deep Sequencing Platform, Max Planck Institute for Heart and Lung Research, 61231 Bad Nauheim, Germany; ^5^ Bioinformatics Core Unit (BCU), Max Planck Institute for Heart and Lung Research, 61231 Bad Nauheim, Germany; ^6^ Centre Hospitalier Universitaire Sainte-Justine Research Center, 3175 Chemin de la Côte-Sainte-Catherine, H3T 1C5 Montréal, QC, Canada; ^7^ Department of Pathology and Cell Biology, Faculty of Medicine, Université de Montréal, H3T 1J4 Montréal, QC, Canada

**Keywords:** Flt1, Vegfa, Zebrafish, Cardiac regeneration, Egr3

## Abstract

VEGFA administration has been explored as a pro-angiogenic therapy for cardiovascular diseases including heart failure for several years, but with little success. Here, we investigate a different approach to augment VEGFA bioavailability: by deleting the VEGFA decoy receptor VEGFR1 (also known as FLT1), one can achieve more physiological VEGFA concentrations. We find that after cryoinjury, zebrafish *flt1* mutant hearts display enhanced coronary revascularization and endocardial expansion, increased cardiomyocyte dedifferentiation and proliferation, and decreased scarring. Suppressing Vegfa signaling in *flt1* mutants abrogates these beneficial effects of *flt1* deletion. Transcriptomic analyses of cryoinjured *flt1* mutant hearts reveal enhanced endothelial MAPK/ERK signaling and downregulation of the transcription factor gene *egr3*. Using newly generated genetic tools, we observe *egr3* upregulation in the regenerating endocardium, and find that Egr3 promotes myofibroblast differentiation. These data indicate that with enhanced Vegfa bioavailability, the endocardium limits myofibroblast differentiation via *egr3* downregulation, thereby providing a more permissive microenvironment for cardiomyocyte replenishment after injury.

## Teaser

**Summary:** *flt1* inactivation promotes zebrafish cardiac regeneration by enhancing coronary revascularization and endocardial expansion, while limiting myofibroblast differentiation.

## INTRODUCTION

Due to the limited regenerative capacity of the adult human heart, myocardial infarction (MI) causes permanent loss of myocardium as well as fibrotic remodeling, ultimately leading to heart failure (McMurray and Pfeffer, 2005; Murry et al., 2006; Laflamme and Murry, 2011; Senyo et al., 2013; Tanai and Frantz, 2015). The formation of new blood vessels after MI is crucial for the survival and function of cardiac tissue. Achieving adequate revascularization has been a primary goal in the field of regenerative therapy for heart disease. However, attempts to promote revascularization and cardiac regeneration by administering pro-angiogenic factors, such as vascular endothelial growth factor A (VEGFA) have thus far had limited success (Lupu et al., 2020).

Contrary to humans, zebrafish possess a remarkable ability to regenerate their heart, wherein lost cardiomyocytes are replenished and fibrosis is resolved post-injury (Poss et al., 2002; Chablais et al., 2011; Gonzalez-Rosa et al., 2011; Bevan et al., 2020). After injury, the zebrafish heart has the innate ability to efficiently revascularize damaged tissues (Marin-Juez et al., 2016, 2019; El-Sammak et al., 2022). Vegfaa regulates coronary revascularization in zebrafish hearts by promoting intra-ventricular vessel sprouting, which, along with superficial sprouting, creates a supportive framework for newly regenerated cardiomyocytes to occupy the injured tissue (Marin-Juez et al., 2019). VEGFA primarily interacts with two transmembrane tyrosine kinases predominantly expressed in endothelial cells: VEGF receptor 1 (VEGFR1; also known as FLT1) and VEGFR2 (de Vries et al., 1992; Millauer et al., 1993). While VEGFR2 is recognized as the primary VEGFA signaling receptor due to its robust kinase activity after binding, FLT1 binds VEGFA with even greater affinity (Roberts et al., 2004), but displays very weak kinase activity (Waltenberger et al., 1994; Ito et al., 1998). Besides the transmembrane form, *FLT1* also encodes a soluble form (sFLT1) that lacks its transmembrane and kinase domains, rendering it incapable of kinase activity even when bound to VEGFA (Shibuya, 2013). Therefore, FLT1 is usually regarded as a decoy receptor that attenuates VEGFR2 signaling by sequestering VEGFA. Previous studies have shown that *Flt1^−/−^* mice display embryonic lethality (Fong et al., 1995), while mice lacking *Flt1* function in endothelial cells exhibit hypervascularization as well as angiogenesis-induced cardiomyocyte growth (Kivela et al., 2019).

Previous studies in zebrafish have shown that constitutive overexpression of Vegfaa can stimulate cardiomyocyte cell cycle re-entry while blocking cardiac regeneration, further highlighting the importance of dose and timing (Karra et al., 2018). More recently, a dose-dependent mitogenic effect of VEGFA on endothelial cells has been reported (Pontes-Quero et al., 2019). We hypothesized that more physiological levels of VEGFA could promote cardiac regeneration. To test this hypothesis and identify pro-regenerative mechanisms, in this study, we investigate cardiac regeneration in *flt1* mutant zebrafish.

*flt1* deletion in zebrafish enhances the response of the cardiac endothelium to cardiac cryoinjury; it also enhances cardiomyocyte regeneration and reduces scarring. Furthermore, *flt1* deletion leads to the downregulation of the transcription factor gene *egr3*. Manipulation of *egr3* levels during cardiac regeneration reveals a role for Egr3 in promoting myofibroblast differentiation. Overall, our data indicate that physiological stimulation of Vegfa signaling boosts cardiac regeneration by (1) enhancing endothelial replenishment, (2) limiting myofibroblast differentiation and (3) reducing scarring. These conditions might create a more-permissive milieu for cardiomyocyte repopulation.

## RESULTS

### *flt1* deletion promotes coronary vascular development as well as endothelial regeneration after cardiac cryoinjury

Previous studies have shown that, in zebrafish larvae, *flt1* is expressed predominantly in endothelial cells and that it plays a pivotal role in modulating angiogenesis and vessel branching morphogenesis (Krueger et al., 2011; Zygmunt et al., 2011). While disrupting the signaling function of the membrane form of Flt1 in zebrafish has no obvious effect on angiogenesis, mutations affecting the function of its soluble form enhance endothelial growth in a Vegfaa-dependent manner (Matsuoka et al., 2016; Wild et al., 2017). Therefore, we hypothesized that Flt1 might also be involved in limiting coronary vessel growth during development and regeneration. To test this hypothesis, we used the *Tg(-0.8flt1:RFP)* line to visualize coronary endothelial cells (cECs) (Bussmann et al., 2010) and crossed it with *flt1* mutants (Matsuoka et al., 2016) and a *Tg(hsp70l:sflt1)* overexpression line (Matsuoka et al., 2016). Coronary formation begins 1-2 months after fertilization (Harrison et al., 2015). We analyzed the coronary network at 42 days post-fertilization (dpf) (body length∼20 mm), a timepoint when coronary vessels begin to form a basic network in wild-type hearts. We found that the coronary network in *flt1* mutants was expanded, with increased branching and vascular coverage over the ventricles (Fig. S1A,B). In contrast, the formation of the coronary network was notably suppressed in juvenile hearts overexpressing *sflt1*, with only a few sprouts around the atrioventricular canal (Fig. S1C,D). While unaffected in *flt1* mutants, ventricular volume was significantly reduced after developmental overexpression of *sflt1* (Fig. S1E). When analyzing ventricles at adult stages, we found no differences in vessel coverage between *flt1^−/−^* and *flt1^+/+^* siblings (Fig. S1F-G).

**Fig. 1.**
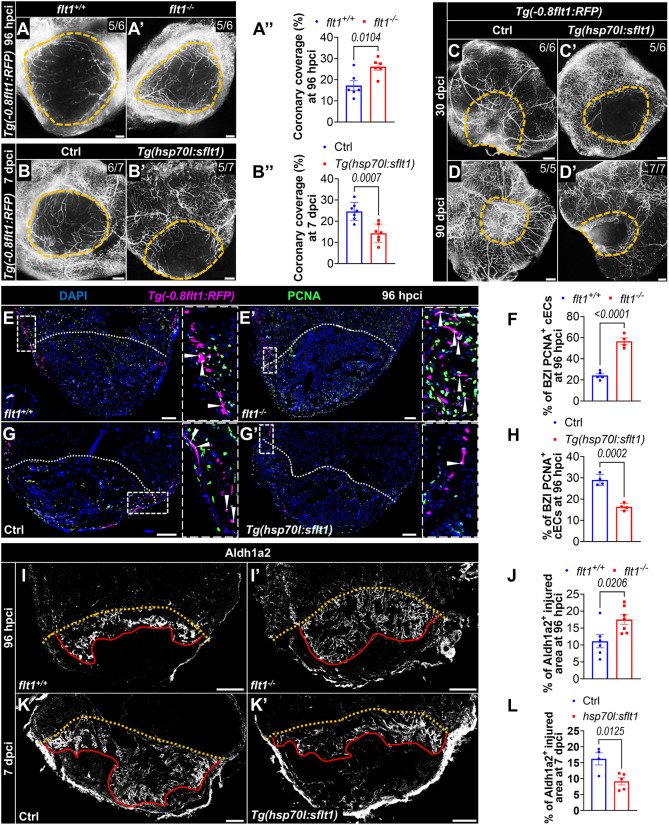
**Flt1 regulates endothelial regeneration.** (A-B″) Whole-mount images of cryoinjured ventricles from *Tg(-0.8flt1:RFP);flt1^+/+^* (*n*=6, A) and *Tg(-0.8flt1:RFP);flt1^−/−^* (*n*=6, A′) zebrafish, and from *Tg(-0.8flt1:RFP)* (Ctrl, *n*=7, B) and *Tg(-0.8flt1:RFP);Tg(hsp70l:sflt1)* (*n*=7, B′) zebrafish showing revascularization of the injured area at 96 hpci (A,A′) and 7 dpci (B,B′), and the corresponding percentage of coronary coverage of the injured area (A″,B″). (C-D′) Whole-mount images of cryoinjured ventricles from *Tg(-0.8flt1:RFP)* (Ctrl, C,D) and *Tg(-0.8flt1:RFP);Tg(hsp70l:sflt1)* (C′,D′) zebrafish showing revascularization at 30 dpci (C,C′) and 90 dpci (D,D′). (E,E′,G,G′) Immunostaining for RFP (cECs, magenta) and PCNA (proliferation marker, green) with DAPI (blue) counterstaining on sections of cryoinjured ventricles from *Tg(-0.8flt1:RFP);flt1^+/+^* (*n*=5, E) and *Tg(-0.8flt1:RFP);flt1^−/−^* (*n*=4, E′) zebrafish, and from *Tg(-0.8flt1:RFP)* (Ctrl, *n*=4, G) and *Tg(-0.8flt1:RFP);Tg(hsp70l:sflt1)* (*n*=4, G′) zebrafish at 96 hpci. Arrowheads indicate PCNA^+^ cECs. Areas outlined are shown at higher magnification on the right. (F,H) Percentage of PCNA^+^ cECs in the border zone and injured area (BZI) of the indicated genotypes at 96 hpci. (I,I′,K,K′) Immunostaining for Aldh1a2 (activated endocardium, white) on sections of cryoinjured ventricles from *flt1^+/+^* (*n*=6, I) and *flt1^−/−^* (*n*=7, I′) zebrafish at 96 hpci, and from non-transgenic Ctrl (*n*=4, K) and *Tg(hsp70l:sflt1)* (*n*=5, K′) zebrafish at 7 dpci. Red lines indicate the extent of activated endocardium in the injured area. (J,L) Percentage of Aldh1a2^+^ injured area from the indicated genotypes. Orange and white dotted lines indicate the injured area. Scale bars: 100 µm. Data are mean±s.e.m. (two-tailed, unpaired Student's *t*-test with *P* values shown in the graphs).

Next, we set out to test whether Flt1 regulates coronary regeneration. To this end, we cryoinjured the ventricles of *flt1* mutants and *Tg(hsp70l:sflt1)* zebrafish in the *Tg(-0.8flt1:RFP)* background. For the *Tg(hsp70l:sflt1)* experiments, we implemented daily heat shock treatments before and after the cryoinjury until the observation timepoints (Fig. S2A), as described in our previous study (El-Sammak et al., 2022). We analyzed coronary coverage of the injured tissue in *flt1* mutants at 96 h post-cryoinjury (hpci), when revascularization of the injury is obvious and coronary endothelial cell proliferation peaks in wild types (Marin-Juez et al., 2016; Ross Stewart et al., 2022). We found that *flt1* mutants exhibited significantly enhanced revascularization of the injured tissue compared with wild types, as measured by coronary vessel coverage (Fig. 1A-A″). In contrast, zebrafish overexpressing *sflt1* exhibited significantly impaired revascularization at 7 days post cryoinjury (dpci) (Fig. 1B-B″), a timepoint when regenerating coronaries fully cover the injured tissue in wild types (El-Sammak et al., 2022). Injured areas in *sflt1*-overexpressing zebrafish were still un-revascularized at 30 and 90 dpci (Fig. 1C-D′). To examine the cECs more closely, we quantified their proliferation in the border zone and injured area (BZI) at 96 hpci and found that it was significantly increased in *flt1* mutants and decreased in *sflt1*-overexpressing zebrafish when compared with wild-type and control siblings, respectively (Fig. 1E-H).

FLT1 serves as a decoy receptor for VEGFA, thereby reducing VEGFA bioavailability for VEGFR2 and attenuating VEGFA/VEGFR2-induced angiogenesis (Ruiz de Almodovar et al., 2009; Ho et al., 2012; Kivela et al., 2014; Robciuc et al., 2016). Indeed, the pro-angiogenic phenotype in *flt1* mutants has been shown to be due to increased Vegfa bioavailability (Matsuoka et al., 2016; Wild et al., 2017). To confirm that the observed phenotypes during cardiac regeneration are Vegf dependent, we overexpressed a dominant-negative form of *vegfaa* (*dnvegfaa*) (Muller et al., 1997a,b; Rossi et al., 2016) in *flt1* mutants using the *Tg(hsp70l:dnvegfaa)* line (Marin-Juez et al., 2016), and quantified coronary coverage of the injured area as well as cEC proliferation at 96 hpci. Overexpression of *dnvegfaa* in *flt1* mutants blocked most of the increase in tissue revascularization and cEC proliferation observed after *flt1* deletion (Fig. S3A-D).

The observed phenotypes in cECs prompted us to investigate whether modulation of *flt1* also affected the behavior of the other cardiac endothelial cell population, the endocardium, given that revascularization is partially regulated by endocardial cues (Marin-Juez et al., 2019). We used Aldh1a2 as a marker of activated endocardial cells (EdCs) (Kikuchi et al., 2011; Munch et al., 2017) and found that *flt1* mutants display an increased expansion of Aldh1a2^+^ cells within the injured area compared with wild types at 96 hpci (Fig. 1I,J). This finding was further corroborated by co-staining for Aldh1a2 and Fli1, and quantifying the coverage of Aldh1a2^+^/Fli1^+^ cells within the injured area (Fig. S2B,C). Given that EdC proliferation is high at 96 hpci (Munch et al., 2017), we also assessed it at this timepoint and found a significant increase within the injured area of *flt1* mutants (Fig. S2D-E). While *sflt1* overexpression strongly reduced cEC proliferation (Fig. 1G-H), it did not appear to affect endocardial expansion at 96 hpci (Fig. S2F-G). However, *sflt1* overexpression reduced endocardial expansion significantly at 7 dpci (Fig. 1K-L). Altogether, these data indicate that increased Vegfa bioavailability enhances coronary regeneration after cardiac cryoinjury in zebrafish. Moreover, increased Vegfa signaling can also enhance endocardial expansion.

### *flt1* modulation alters cardiomyocyte regeneration and scarring after cardiac cryoinjury

**Fig. 2.**
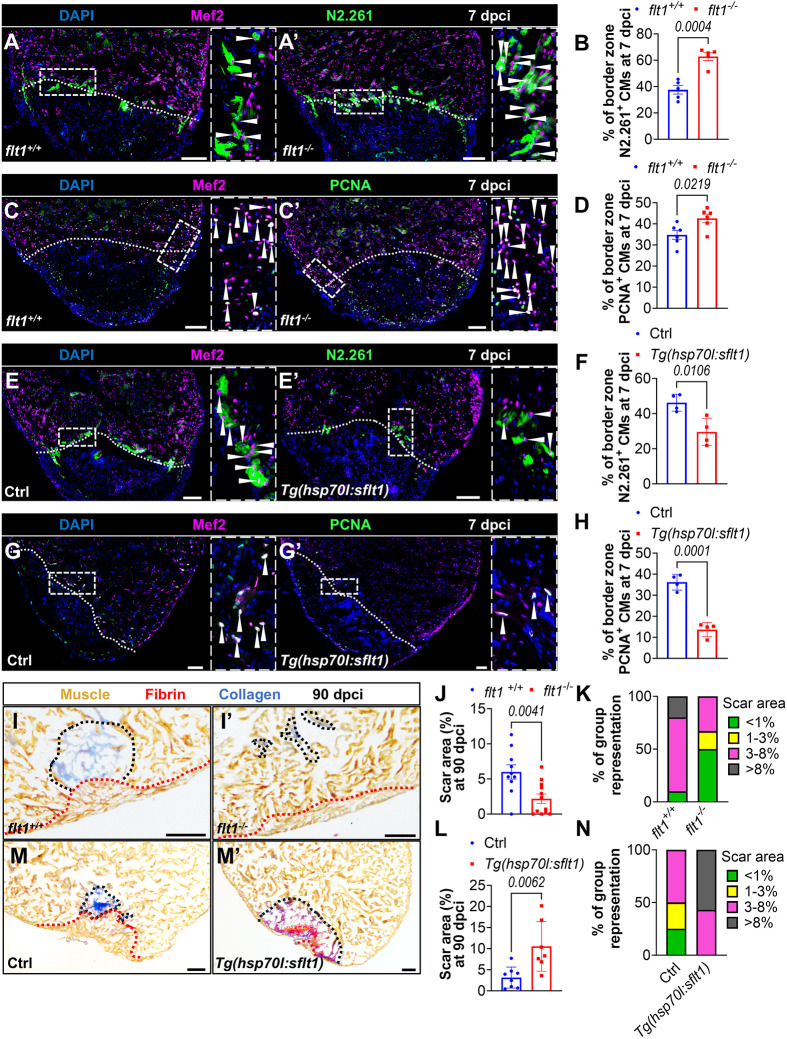
***flt1* modulation alters cardiomyocyte regeneration and scarring after cardiac cryoinjury.** (A,A′,E,E′) Immunostaining for Mef2 (CM nuclei, magenta) and N2.261 (embryonic myosin heavy chain, green) with DAPI (blue) counterstaining on sections of cryoinjured ventricles from *flt1^+/+^* (*n*=5, A) and *flt1^−/−^* (*n*=5, A′) zebrafish, and from non-transgenic Ctrl (*n*=4, E) and *Tg(hsp70l:sflt1)* (*n*=4, E′) zebrafish at 7 dpci. Arrowheads indicate N2.261^+^ CMs. Areas outlined are shown at higher magnification on the right. White dotted lines indicate the injured area. (B,F) Percentage of N2.261^+^ CMs in the border zone of the indicated genotypes at 7 dpci. (C,C′,G,G′) Immunostaining for Mef2 (CM nuclei, magenta) and PCNA (green) with DAPI (blue) counterstaining on sections of cryoinjured ventricles from *flt1^+/+^* (*n*=6, C) and *flt1^−/−^* (*n*=6, C′) zebrafish, and from non-transgenic Ctrl (*n*=4, G) and *Tg(hsp70l:sflt1)* (*n*=4, G′) zebrafish at 7 dpci. Arrowheads indicate PCNA^+^ CMs. Areas outlined are shown at higher magnification on the right. White dotted lines indicate the injured area. (D,H) Percentage of PCNA^+^ CMs in the border zone of the indicated genotypes at 7 dpci. (I,I′,M,M′) AFOG staining on sections of cryoinjured ventricles from *flt1^+/+^* (*n*=10, I) and *flt1^−/−^* (*n*=12, I′) zebrafish, and from non-transgenic Ctrl (*n*=8, M) and *Tg(hsp70l:sflt1)* (*n*=7, M′) zebrafish at 90 dpci. Orange, muscle; red, fibrin; blue, collagen. Black and red dotted lines delineate the scar and regenerated muscle wall areas, respectively. (J,L) Percentage of scar area relative to ventricular area in the indicated genotypes at 90 dpci. (K,N) Graphs showing the representation of groups of different scar area sizes in the indicated genotypes at 90 dpci. Scale bars: 100 µm. Data are mean±s.e.m. (two-tailed, unpaired Student's *t*-test with *P* values shown in the graphs).

During cardiac regeneration in zebrafish, cardiomyocytes undergo dedifferentiation and proliferation (Kikuchi et al., 2010; Morikawa et al., 2015; Beisaw et al., 2020; Tsedeke et al., 2021), processes that are regulated, at least in part, by cECs (Marin-Juez et al., 2016, 2019) and EdCs (Kikuchi et al., 2011; Munch et al., 2017; Galvez-Santisteban et al., 2019; Zhao et al., 2019). In view of the endothelial phenotypes observed upon manipulation of *flt1* function, we sought to investigate its impact on cardiomyocyte regeneration. Using the embryonic myosin heavy chain antibody N2.261 as a readout for cardiomyocyte dedifferentiation (Sallin et al., 2015), and the DNA replication marker PCNA as an indicator of cardiomyocyte proliferation, we assessed the percentage of dedifferentiating and proliferating border zone cardiomyocytes in *flt1*^−/−^ and *Tg(hsp70l:sflt1)* zebrafish after cardiac cryoinjury. We observed a marked increase in both cardiomyocyte dedifferentiation and proliferation in *flt1* mutants at 96 hpci and 7 dpci (Fig. 2A-D, Fig. S4A-D). Notably, cardiomyocyte proliferation in *flt1* mutants at 96 hpci was comparable to that observed in *flt1* mutants at 7 dpci (Fig. 2D and Fig. S4C-D), when cardiomyocyte proliferation is at its highest in wild types, suggesting a change in cardiomyocyte proliferation dynamics. In contrast, cardiomyocyte dedifferentiation and proliferation were significantly reduced in *sflt1*-overexpressing zebrafish when compared with controls (Fig. 2E-H). Together, these results indicate that *flt1* negatively regulates cardiomyocyte dedifferentiation and proliferation during cardiac regeneration in zebrafish. We also found that the increase in cardiomyocyte proliferation observed in *flt1* mutants was blocked upon *dnvegfaa* overexpression (Fig. S3E-F).

Given the impact of *flt1* modulation on endothelial and cardiomyocyte regeneration, we explored whether these alterations affected scarring after cardiac cryoinjury. In line with the other phenotypes, we observed significantly smaller scar areas at 90 dpci in *flt1* mutants when compared with wild-type siblings (Fig. 2I-K). Conversely, ventricles overexpressing *sflt1* failed to regenerate, as evidenced by the presence of large fibrin-rich scars at both 30 and 90 dpci (Fig. 2M,N, Fig. S4E-G), consistent with observations from whole-mount samples (Fig. 1C′,D′). In contrast, wounds in control cryoinjured ventricles displayed a continuous myocardial wall enclosing the injury with collagen-rich scars at 30 dpci and a limited scar area at 90 dpci (Fig. 2M, Fig. S4E). Collectively, these findings indicate that *flt1* limits myocardial repopulation and increases scarring during cardiac regeneration.

### *flt1* deletion causes the upregulation of endothelial MAPK/ERK signaling and the downregulation of *egr3* expression during cardiac regeneration

**Fig. 3.**
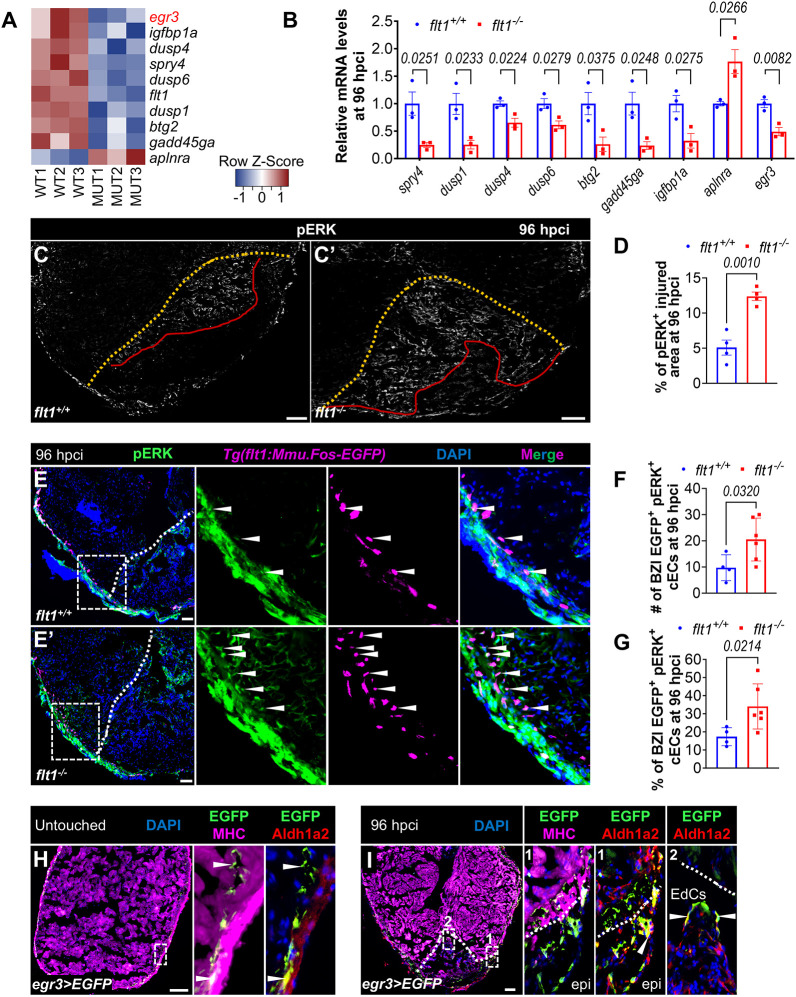
***flt1* deletion causes the upregulation of endothelial MAPK/ERK signaling and the downregulation of *egr3* expression during cardiac regeneration.** (A,B) Heat-map (A) and RT-qPCR validation (B) showing differentially regulated genes of interest in the border zone and injured area (BZI) of cryoinjured ventricles from *flt1^+/+^* and *flt1^−/−^* zebrafish at 96 hpci. (C,C′) Immunostaining of cryoinjured ventricles from *flt1^+/+^* (*n*=4, C) and *flt1^−/−^* (*n*=4, C′) zebrafish at 96 hpci for pERK (phosphorylated ERK, white). Red lines indicate the range of pERK^+^ injured area. Dotted orange line indicates the injured area. (D) Quantification of the percentage of pERK^+^ injured area in the indicated genotypes at 96 hpci. (E,E′) Immunostaining for pERK (green) and EGFP (cECs, magenta) with DAPI (blue) counterstaining on sections of cryoinjured ventricles from *Tg(flt1:Mmu.Fos-EGFP);flt1^+/+^* (*n*=4, E) and *Tg(flt1:Mmu.Fos-EGFP);flt1^−/−^* (*n*=6, E′) zebrafish at 96 hpci. Arrowheads indicate pERK^+^ cECs in the BZI. Areas outlined are shown at higher magnification on the right. (F,G) Quantification showing the number (F) and percentage (G) of pERK^+^ cECs in the BZI of the indicated genotypes at 96 hpci. (H,I) Immunostaining for EGFP (green), MHC (myosin heavy chain, magenta) and Aldh1a2 (red) with DAPI (blue) counterstaining on representative sections from untouched (H) and cryoinjured (I) *Pt(egr3:Gal4-VP16);Tg(5xUAS:EGFP)* (abbreviated as *egr3>EGFP*) ventricles at 96 hpci. Arrowheads indicate EGFP*^+^* cells. Areas outlined are shown at higher magnification on the right. White dotted lines indicate the injured area. epi, epicardium. Scale bars: 100 µm. Data are mean±s.e.m. (two-tailed, unpaired Student's *t*-test with *P* values shown in the graphs).

To gain further insight into how *flt1* regulates cardiac regeneration, we conducted transcriptional analysis of the BZI from *flt1^+/+^* and *flt1^−/−^* sibling zebrafish at 96 hpci (Fig. S5A). We identified 85 differentially expressed genes (DEGs) (*padj*<0.05), with 72 of them showing downregulation in *flt1* mutants (Fig. S5B,C). Gene Ontology and KEGG pathway analyses revealed a significant downregulation of genes associated with negative regulation of MAPK/ERK signaling (Fig. S5D). Further investigation of the DEGs within these categories revealed a decrease in the expression of genes encoding MAPK/ERK antagonists, including *spry4*, *dusp1*, *dusp4*, and *dusp6* in cryoinjured *flt1* mutant ventricles, as well as a decrease in the expression of multiple anti-proliferative factor genes such as *btg2*, *gadd45ga* and *igfbp1a*. Additionally, we observed an upregulation of the angiogenic factor gene *aplnra* among the few upregulated genes in cryoinjured *flt1* mutant ventricles. Changes in expression levels of these genes were confirmed by RT-qPCR analysis (Fig. 3A,B).

Previous studies have revealed a role for MAPK/ERK signaling in zebrafish cardiac regeneration, and its activation in the injured endocardium (Liu and Zhong, 2017; Tahara et al., 2021; Cardeira-da-Silva et al., 2024). We speculated that the downregulation of MAPK/ERK antagonists might facilitate MAPK/ERK activation in endocardial cells after cardiac cryoinjury. After injury, MAPK/ERK signaling is enhanced in the activated endocardium, as assessed by phospho-ERK (pERK) immunostaining (Fig. S5E) (Cardeira-da-Silva et al., 2024). Notably, we observed a significant expansion of pERK^+^ endocardium within the injured area in *flt1* mutants compared with wild-type siblings (Fig. 3C-D). By using the *Tg(flt1:Mmu.Fos-EGFP)* line (Nicenboim et al., 2015; El-Sammak et al., 2022) to label specifically the coronary endothelium, we also observed a significant increase in both the number and percentage of pERK^+^ cECs at the border zone in cryoinjured *flt1* mutant ventricles (Fig. 3E-G′). Additionally, *flt1* mutants exhibited a higher abundance of coronary vessels at the border zone (Fig. 3E′), consistent with a previous report (Marin-Juez et al., 2019). Taken together, these findings indicate that *flt1* deletion enhances endothelial MAPK/ERK signaling after cardiac cryoinjury.

Interestingly, one of the most downregulated genes in cryoinjured *flt1* mutant ventricles was *early growth response 3* (*egr3*) (Fig. 3A,B, Fig. S5C). To determine the expression pattern of *egr3* after cardiac cryoinjury in zebrafish, we used the recently generated knock-in *egr3* Gal4 line *Pt(egr3:Gal4-VP16)* (da Silva et al., 2024) in conjunction with the *Tg(5xUAS:EGFP)* (Asakawa et al., 2008), together abbreviated as *egr3>EGFP*. Immunostaining for GFP, MHC and Aldh1a2 revealed that, in untouched ventricles, *egr3>*EGFP is only marginally expressed between the cortical and trabecular myocardial layers, as well as in the epicardium (Fig. 3H). After cryoinjury, *egr3>*EGFP expression was induced in the injured area, characterized by broader expression within and alongside the epicardium-derived cells (EPDCs) that cover the injured tissue, as well as in the Aldh1a2^+^ EdCs that expand into the wound (Fig. 3I).

### *flt1* deletion limits myofibroblast differentiation and promotes cardiomyocyte repopulation by downregulating *egr3*

**Fig. 4.**
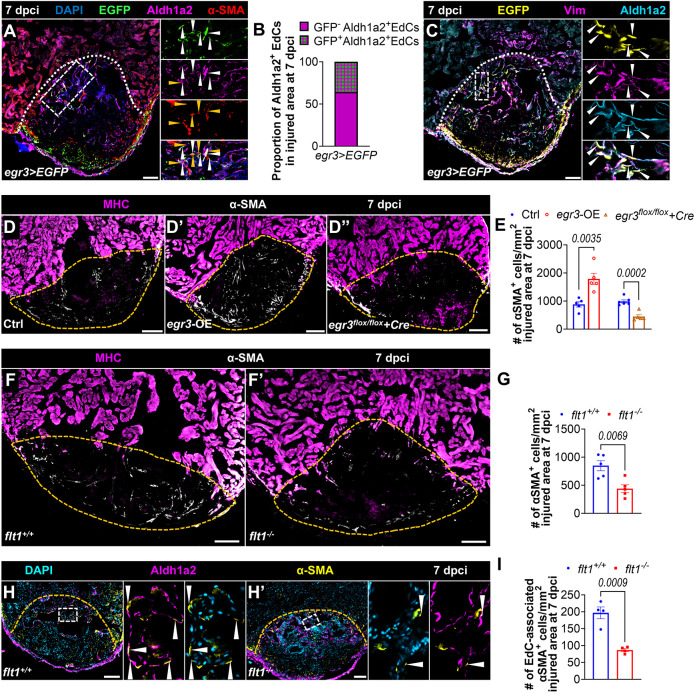
***flt1* deletion limits myofibroblast differentiation.** (A) Immunostaining for EGFP (green), Aldh1a2 (magenta) and α-SMA (myofibroblasts, red) with DAPI (blue) counterstaining on representative sections of cryoinjured *egr3>EGFP* ventricles at 7 dpci. White and orange arrowheads indicate the EGFP^+^Aldh1a2^+^ EdCs and α-SMA^+^ cells, respectively. (B) Proportion of EGFP^+^ EdCs within the wound of cryoinjured *egr3>EGFP* ventricles (*n*=3) at 7 dpci (see Table S4 for raw data). (C) Immunostaining for GFP (yellow), Aldh1a2 (cyan) and Vim (fibroblasts/myofibroblasts, magenta) with DAPI (blue) counterstaining on representative sections of cryoinjured *egr3>EGFP* ventricles at 7 dpci. Arrowheads indicate the EGFP^+^Aldha1a^+^Vim^+^ EdCs. (D-D″,F,F′) Immunostaining for MHC (magenta) and α-SMA (white) on sections of cryoinjured ventricles from Ctrl [*n*=5 for *egr3* OE, *n*=6 for *Tg(hsp70l:Cre);egr3^flox/flox^*, D], *egr3* OE (*n*=5, D′) and *Tg(hsp70l:Cre);egr3^flox/flox^* (*n*=5, D″) zebrafish, and from *flt1^+/+^* (*n*=5, F) and *flt1^−/−^* (*n*=5, F′) zebrafish at 7 dpci. (E,G) Quantification of α-SMA^+^ cell number within the wound of the indicated genotypes at 7 dpci. (H,H′) Immunostaining for Aldh1a2 (magenta) and α-SMA (yellow) with DAPI (cyan) counterstaining on sections of cryoinjured *flt1^+/+^* (*n*=4, H) and *flt1^−/−^* (*n*=4, H′) ventricles at 7 dpci. Arrowheads indicate endocardial-associated α-SMA^+^ cells. (I) Quantification of endocardial-associated α-SMA^+^ cell number within the wound on ventricular sections of the indicated genotypes at 7 dpci. White and orange dotted lines indicate the injured area. Areas outlined are shown at higher magnification on the right in A,C,H and H′. Scale bars: 100 µm. Data are mean±s.e.m. (two-tailed, unpaired Student's *t*-test with *P* values shown in the graphs).

We noted that the *egr3>*EGFP expression pattern in both untouched and cryoinjured ventricles closely resembled the distribution of fibroblasts (Sanchez-Iranzo et al., 2018). To better define the different cell types expressing *egr3* before and after cardiac cryoinjury, we analyzed a published single-cell RNA sequencing dataset (Koth et al., 2020). This analysis revealed that after cryoinjury, *egr3* is upregulated in certain endothelial cell clusters, as well as in fibroblasts and myofibroblasts (Fig. S6A-D). We then conducted immunostaining on cryoinjured *egr3>EGFP* ventricles at 7 dpci for EGFP, Aldh1a2 and α-SMA, and observed that about 36% of Aldh1a2^+^ EdCs expressed *egr3>*EGFP, with these EdCs being positive for α-SMA or located in close proximity to α-SMA^+^ cells (Fig. 4A,B, Table S4). To further characterize these *egr3>*EGFP-expressing EdCs, we used vimentin (Vim) as a marker for fibroblasts/myofibroblasts and found that they were indeed positive for Vim (Fig. 4C). These data are indicative of the transition of these EdCs towards a fibroblast/myofibroblast identity, consistent with the reanalysis results of the single-cell RNA sequencing dataset. Altogether, these data led us to hypothesize that *egr3* promotes endothelial-to-mesenchymal transition (EndoMT) during zebrafish heart regeneration.

EndoMT is a process during which endothelial cells lose their identity and give rise to mesenchymal cells such as fibroblasts and myofibroblasts, contributing to organ fibrosis, including cardiac fibrosis (Kovacic et al., 2019; Piera-Velazquez and Jimenez, 2019). An increase in EndoMT leads to mammalian-like fibrosis after cardiac cryoinjury in zebrafish (Allanki et al., 2021). Myofibroblast differentiation is a hallmark of mammalian-like fibrosis post cardiac injury (Micallef et al., 2012; Davis and Molkentin, 2014). Notably, increased *Egr3* expression promotes pro-fibrotic responses in scleroderma and leads to myofibroblast accumulation in lesional dermis (Fang et al., 2013). To investigate whether Egr3 plays a role in myofibroblast differentiation during zebrafish cardiac regeneration, we leveraged recently developed gain- and loss-of-function genetic tools (da Silva et al., 2024). Specifically, we used an *egr3* overexpression line *Tg(hsp70l:Gal4);Tg(5xUAS:egr3)*, abbreviated as *egr3*-OE, and an *egr3* floxed line crossed to an *hsp70l:Cre* line (Fig. S7A). We subjected these adult zebrafish along with their control siblings to cardiac cryoinjury and heat-shock treatments (Fig. S7A,B), and analyzed mRNA levels (Fig. S7C,D). To assess myofibroblast differentiation, we stained *egr3*-OE ventricles and controls for α-SMA expression at 7 dpci, and found an increased number of α-SMA^+^ cells in the injured area after *egr3* OE (Fig. 4D,D′,E). Conversely, ventricles from heat-shocked *Tg(hsp70l:Cre);egr3^flox/flox^* (abbreviated as *egr3^flox/flox^+Cre*) and *Tg(hsp70l:Cre);egr3^flox/+^* (abbreviated as *egr3^flox/+^+Cre*) zebrafish displayed a significant decrease of α-SMA^+^ cells in the injured area compared with controls (Fig. 4D,D″,E, Fig. S7E-F), altogether suggesting a role for *egr3* in promoting myofibroblast differentiation.

Since *egr3* was downregulated in cryoinjured *flt1*^−/−^ hearts, we quantified the number of α-SMA positive cells in these ventricles and found a significant reduction (Fig. 4F-G), consistent with the reduced scarring observed in these animals. As *egr3* is upregulated in the regenerating endocardium, we also quantified endocardial-associated α-SMA^+^ myofibroblasts, which we define as myofibroblasts colocalized with, in morphological continuity with or in close proximity to EdCs. In agreement with the decrease in the total number of α-SMA^+^ myofibroblasts within the injured tissue, the number of endocardial-associated α-SMA^+^ myofibroblasts more than halved in cryoinjured *flt1^−/−^* hearts (Fig. 4H-I). Altogether, these data indicate that *flt1^−/−^* hearts exhibit a hyper-invasive endocardial phenotype as well as reduced endocardial-associated myofibroblast differentiation, the latter likely due to the downregulation of *egr3*.

**Fig. 5.**
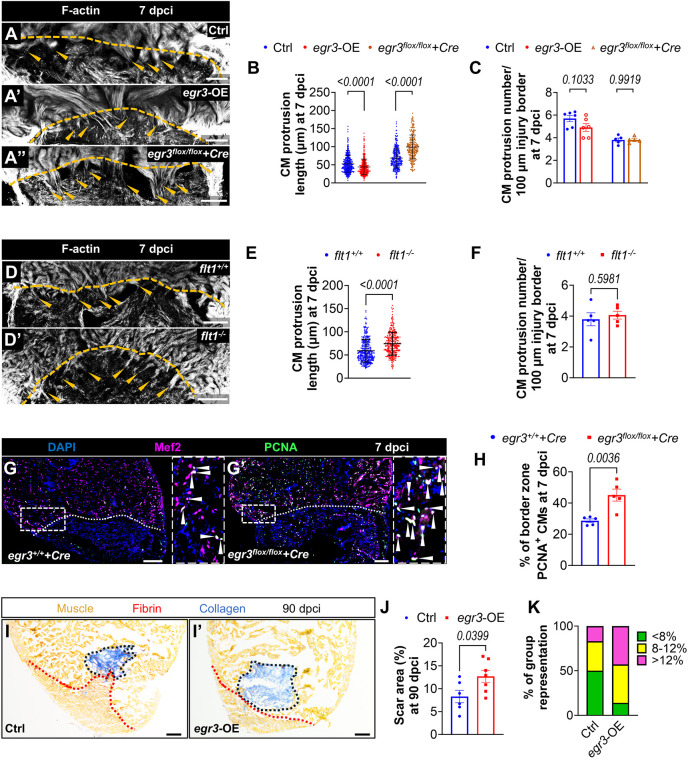
***flt1* deletion promotes cardiomyocyte repopulation by downregulating *egr3*.** (A-A″) Phalloidin staining for F-actin (white) on 50 μm sections of cryoinjured ventricles from Ctrl [*n*=6 for *egr3* OE, *n*=5 for *Tg(hsp70l:Cre);egr3^flox/flox^*, A], *egr3* OE (*n*=6, A′) and *Tg(hsp70l:Cre);egr3^flox/flox^* (*n*=5, A″) zebrafish at 7 dpci. Orange arrowheads indicate the protruding CMs in the injured area; orange dotted lines indicate the injured area. (B,C) Quantification of CM protrusion length (B) and number (C) in the indicated genotypes at 7 dpci. (D,D′) Phalloidin staining for F-actin (white) on 50 μm sections of cryoinjured ventricles from *flt1^+/+^* (*n*=5, D) and *flt1^−/−^* (*n*=5, D′) zebrafish at 7 dpci. Orange arrowheads indicate the protruding CMs in the injured area; orange dotted lines indicate the injured area. (E,F) Quantification of CM protrusion length (E) and number (F) in the indicated genotypes at 7 dpci. (G,G′) Immunostaining for Mef2 (CM nuclei, magenta) and PCNA (green) with DAPI (blue) counterstaining on sections of cryoinjured ventricles from *Tg(hsp70l:Cre);egr3^+/+^* (*n*=5, G) and *Tg(hsp70l:Cre);egr3^flox/flox^* (*n*=5, G′) sibling zebrafish at 7 dpci. Arrowheads indicate PCNA^+^ CMs. Areas outlined are shown at higher magnification on the right. (H) Percentage of PCNA^+^ CMs in the border zone of the indicated genotypes at 7 dpci. (I,I′) AFOG staining on sections of cryoinjured non-transgenic Ctrl (*n*=6, I) and *egr3* OE (*n*=7, I′) ventricles at 90 dpci. Orange, muscle; red, fibrin; blue, collagen. Black and red dotted lines delineate the scar and regenerated muscle wall areas, respectively. (J) Percentage of scar area relative to ventricular area in the indicated genotypes at 90 dpci. (K) Graphs showing the representation of groups of different scar area sizes in the indicated genotypes at 90 dpci. Scale bars: 100 µm. Data in C,F,H,J are mean±s.e.m. (two-tailed, unpaired Student's *t*-test with *P* values shown in the graphs). Data in B,E are mean±s.d. (two-tailed, Mann–Whitney *U* test with *P* values shown in the graphs).

Myofibroblasts are responsible for the production and deposition of extracellular matrix (ECM) proteins. ECM molecules can regulate cardiomyocyte mobilization and proliferation by directly signaling to cardiomyocytes or altering the microenvironment (Chablais and Jazwinska, 2012; Fang et al., 2013; Wang et al., 2013; Chen et al., 2016; Bassat et al., 2017; Notari et al., 2018; Koth et al., 2020; Wu et al., 2020). Previous studies have shown that ECM deposition strongly influences cardiomyocyte repopulation in injured zebrafish hearts (Wang et al., 2013; Allanki et al., 2021; Constanty et al., 2024 preprint). Therefore, we reasoned that alterations in *egr3* expression might affect cardiomyocyte repopulation after cardiac cryoinjury. To investigate this possibility, we quantified cardiomyocyte protrusions in cryoinjured ventricles at 7 dpci and observed that they were significantly shorter in *egr3*-OE ventricles and significantly longer in *Tg(hsp70l:Cre);egr3^flox/flox^* (Fig. 5A-B) and *Tg(hsp70l:Cre);egr3^flox/+^* (Fig. S7G-H) ventricles compared with their respective control siblings. The number of cardiomyocyte protrusions remained similar across all groups (Fig. 5C, Fig. S7I). Cardiomyocyte protrusion length was also increased in *flt1^−/−^* ventricles at 7 dpci (Fig. 5D,E), while their number was not affected (Fig. 5F). Additionally, we observed a significant increase in cardiomyocyte proliferation in *Tg(hsp70l:Cre);egr3^flox/flox^* (Fig. 5G-H) and *Tg(hsp70l:Cre);egr3^flox/+^* ventricles (Fig. S7J-K), one similar to that observed in *flt1^−/−^* ventricles (Fig. 2C-D). Although cardiomyocyte proliferation in *egr3*-OE ventricles appeared reduced compared with control siblings, the difference was not statistically significant (Fig. S7L-M). To assess whether *egr3*-OE impacted scarring, we performed AFOG staining at 90 dpci. As expected from the previous results, scar area was significantly bigger in *egr3*-OE ventricles than in control siblings (Fig. 5I-K). Altogether, these findings suggest that *egr3* exerts an inhibitory effect on cardiomyocyte repopulation, potentially by modulating myofibroblast-mediated ECM deposition, thereby altering the microenvironment for cardiomyocyte mobilization and proliferation. Collectively, our results indicate that *flt1* deletion restricts myofibroblast differentiation and promotes cardiomyocyte repopulation by downregulating endocardial *egr3*.

## DISCUSSION

Here, we report that *flt1* inactivation enhances zebrafish cardiac regeneration by augmenting the endothelial response, consequently promoting cardiomyocyte regeneration and limiting scarring. FLT1 serves as a decoy receptor for VEGFA, VEGFB and placental growth factor (PGF) (Ruiz de Almodovar et al., 2009). Previous studies in zebrafish embryos and larvae have shown that the *flt1* mutant endothelial phenotypes are Vegfaa dependent (Wild et al., 2017). Moreover, overexpression of *vegfba* and *pgfb* has no impact on vessel sprouting (Klems et al., 2020). Here we used a mutated form of Vegfaa that has been shown to interfere with its affinity for Vegfr2 (Muller et al., 1997a,b; Marin-Juez et al., 2016; Rossi et al., 2016) and found that the *flt1* mutant regeneration phenotypes were rescued, indicating that the enhanced regeneration observed in *flt1* mutants is due to increased Vegfa bioavailability.

Both coronary and endocardial regeneration have been shown to be crucial to support cardiac tissue replenishment in regenerative and non-regenerative organisms (Miquerol et al., 2015; DeBenedittis et al., 2022; Dube et al., 2017; Lai et al., 2017; Tang et al., 2018; Apte et al., 2019; Das et al., 2019; Kivela et al., 2019). In addition, global deletion of *Flt1* at postnatal stages has been shown to increase angiogenesis in different vascular beds and reduce the infarct size in adult mice after ligation of the left anterior descending artery (Ho et al., 2012). Our results further highlight the importance of revascularization for baseline regeneration and the requirement of an augmented endocardial response to enhance it. Moreover, the endocardium serves as a source of Vegfaa (Karra et al., 2018), which regulates intraventricular coronary sprouting in a process termed coronary-endocardial anchoring (Karra et al., 2018; Marin-Juez et al., 2019). Coronary vessels form a vascular scaffold to guide cardiomyocyte development and regeneration (Marin-Juez et al., 2019; DeBenedittis et al., 2022). Therefore, enhancing coronary-endocardial anchoring might itself facilitate cardiomyocyte replenishment in *flt1* mutants.

Our data show that MAPK/ERK signaling was increased in the regenerating endocardium in *flt1* mutants. MAPK/ERK signaling regulates cell proliferation, growth and migration (Unal et al., 2017; Lavoie et al., 2020), and it promotes Vegfa-dependent angiogenesis in zebrafish (Shin et al., 2016). *dusp6*, which encodes a phosphatase that inhibits ERK signaling (Muda et al., 1996; Marchetti et al., 2005), is expressed in cardiac endothelial cells after injury, and zebrafish *dusp6* mutants display improved heart regeneration (Missinato et al., 2018). Moreover, stimulation of MAPK signaling has been shown to stimulate endothelial cell proliferation while inhibiting TGFβ-induced EndoMT (Ichise et al., 2014). Thus, it is reasonable to hypothesize that increased Vegfa signaling in cryoinjured *flt1^−/−^* hearts limits endocardial EndoMT via increased MAPK/ERK activation. Indeed, we find a reduced number of endocardial α-SMA^+^ myofibroblasts in injured *flt1^−/−^* ventricles, further supporting this possibility.

Myofibroblasts contribute to fibrosis by depositing ECM. Changes in ECM composition can influence cardiomyocyte regeneration (Bassat et al., 2017; Mukherjee et al., 2021). ECM-bound proteins can signal directly to cardiomyocytes and/or determine the physical properties of the microenvironment, thereby impacting cardiomyocyte mobilization and division (Chablais and Jazwinska, 2012; Fang et al., 2013; Wang et al., 2013; Chen et al., 2016; Bassat et al., 2017; Notari et al., 2018; Koth et al., 2020; Wu et al., 2020). Fibrosis is the main cause of heart failure after MI in non-regenerative models. However, fibrosis also develops transiently in the regenerating zebrafish heart, where ablation of collagen producing cells impairs cardiomyocyte proliferation (Sanchez-Iranzo et al., 2018). By manipulating its expression, we show that Egr3 negatively regulates myocardial protrusion and proliferation, likely due to the effect of these manipulations on myofibroblast differentiation. It is interesting to note that *egr3* is upregulated as part of the regeneration program deployed by the cryoinjured zebrafish heart. However, its downregulation in conditions of increased Vegfa bioavailability leads to improved regeneration. *EGR3*/EGR3 expression is induced by TGFβ signaling in normal human skin fibroblasts (Fang et al., 2013). TGFβ signaling is induced after cardiac injury in zebrafish and its inhibition is detrimental to heart regeneration, suggesting the need for transient fibrosis as part of a successful cardiac regeneration program (Chablais and Jazwinska, 2012; Sanchez-Iranzo et al., 2018). It is possible that by reducing *egr3* expression, the effect of TGFβ signaling activation is partially attenuated, limiting excessive myofibroblast differentiation and ECM deposition while conserving key components of the fibrotic response. These conditions might create a more-permissive microenvironment that further promotes regenerative processes such us cardiomyocyte dedifferentiation, protrusive behavior and proliferation. Overall, our data indicate that Vegfa-induced attenuation of the fibrotic response enhances cardiac regeneration, at least in part, by providing a more permissive milieu for cardiomyocyte replenishment.

## MATERIALS AND METHODS

### Zebrafish strains and husbandry

Zebrafish larvae were raised under standard conditions. Adult fish were maintained in 3.5 l tanks at a stock density of 10 fish/l with the following parameters: water temperature, 27-27.5°C; light:dark cycle, 14:10 h; pH, 7.0-7.5; conductivity, 750-800 µS/cm. Zebrafish were fed three to five times per day, depending on age, with granular and live food (*Artemia salina*). Health monitoring was carried out twice a year. All procedures performed on animals conform to the guidelines from Directive 2010/63/EU of the European Parliament on the protection of animals used for scientific purposes and were approved by the Animal Protection Committee (Tierschutzkommission) of the Regierungspräsidium Darmstadt (reference: B2/1218 and B2/2058).

To assess coronary development, 28 dpf zebrafish, measuring 14-15 mm in length, were subjected to daily heat shocks until 42 dpf. For studying cardiac regeneration, 3- to 10-month-old male and female zebrafish measuring 27-28 mm in length were used for cardiac cryoinjuries. We used the previously published zebrafish lines *flt1^bns29^* (Matsuoka et al., 2016), *Tg(hsp70l:sflt1)^bns80^* (Matsuoka et al., 2016), *Tg(hsp70l:vegfaa121-F17A)^bns100^* (Marin-Juez et al., 2016), *Tg(-0.8flt1:RFP)^hu5333^* (Bussmann et al., 2010), *Tg(flt1:Mmu.Fos-EGFP)^wz2^* (Nicenboim et al., 2015), *Pt(egr3:Gal4-VP16)^bns576^* (da Silva et al., 2024), *Tg(5xUAS:egr3-p2a-dTomato)^bns607^* (da Silva et al., 2024), *Pt(egr3:loxP-egr3-loxP)^bns661^* (da Silva et al., 2024), *Tg(5xUAS:EGFP)^nkuasgfp1a^* (Asakawa et al., 2008), *Tg(hsp70l:Cre)^zdf13^* (Le et al., 2007) and *Tg(hsp70l:Gal4)^kca4^* (Scheer et al., 2001). For *flt1^−/−^*, sibling *flt1^+/+^* from the same clutch were used as controls; for other transgenic lines, non-transgenic siblings from the same clutch were used as controls. The recombination validation and genotyping of the floxed *egr3* allele were performed as described previously (da Silva et al., 2024). The *Pt(egr3:Gal4-VP16)^bns576^* line is a knock-in line (da Silva et al., 2024); however, no obvious regeneration phenotype was observed in the hearts shown in Fig. 3H,I.

### Cardiac cryoinjury and heat-shock treatment

Cardiac cryoinjury was executed in adult zebrafish hearts following established protocols (Gonzalez-Rosa et al., 2011; Chablais et al., 2011). Before the procedure, adult zebrafish were anesthetized in standard E3 media containing 0.4% tricaine. A small incision was carefully made in the chest area to expose the heart, followed by the application of a cryoprobe precooled in liquid nitrogen to the ventricular apex, maintaining contact for 25 s. Subsequently, cryoinjured fish were gently transferred into fresh system water and allowed to recover. Heat-shock treatments were administered to all zebrafish with the *hsp70 l* promoter by housing them in preheated system water (39°C) for 1 h every 12 or 24 h. Juvenile zebrafish received heat-shock treatments from 28 until 42 dpf at 24 h intervals, while adult zebrafish underwent heat-shock treatments every 12 h, commencing 1 day before cryoinjury and continuing until the designated observation time point post-cryoinjury. Following each heat-shock treatment, the zebrafish were transferred to the facility with water maintained at 28°C.

### Histological analyses, imaging and quantification

Zebrafish hearts were fixed in 4% paraformaldehyde (PFA) for 1 h at room temperature, followed by overnight preservation in a 30% (w/v) sucrose solution prepared in 1× PBS at 4°C. Subsequently, heart samples were embedded in OCT (Tissue-Tek) and stored at −80°C until further processing. Cryosections of 8 and 50μm in thickness were collected on SuperFrost Plus slides (Thermo Fisher Scientific) using a Leica CM1950 cryostat and thereafter stored at −20°C for subsequent use. For immunofluorescence staining of 8 μm cryosections, slides were defrosted for 15 min at room temperature, washed twice with PBST (0.1% Triton X-100 in 1× PBS) and twice with deionized H_2_O to remove OCT, and permeabilized with PBSTx (0.5% Triton X-100 in 1× PBS) for 20 min at room temperature. Cryosections were then incubated in blocking solution [1×PBS, 2% (v/v) goat serum, 0.2% Triton X-100 and 1% DMSO] for 30 min to 1 h at room temperature, followed by incubation with primary antibodies in blocking solution overnight at 4°C. The sections were then rinsed three times with PBST and incubated with secondary antibodies in blocking solution for 2 h at room temperature. Sections were washed another three times in PBST, incubated in 4′,6-diamidino-2-phenylindole (DAPI) (1:10,000, Sigma-Aldrich) for 5 min, and mounted with fluorescence mounting medium (S3023, Agilent Dako). Mef2, N2.261 and pERK immunostainings included an additional step after OCT removal, which involved antigen retrieval in a solution of 10 mM sodium citrate buffer with 0.05% (v/v) Tween 20 (Sigma-Aldrich) adjusted to pH 6.0 for 7 min at 95°C. For immunofluorescence staining of 50 μm cryosections used for the cardiomyocyte protrusion experiments, sections were air-dried at room temperature for 1 h before being washed twice in PBST and subsequently permeabilized in PBSTx for 2 h at room temperature. Sections were then incubated in blocking solution and F-actin was stained with Alexa Fluor-Phalloidin (Thermo Fisher Scientific) at 1:200 dilution overnight at 4°C. After staining, sections were washed three times in PBST and then mounted in fluorescence mounting medium (S3023, Agilent Dako). AFOG staining was performed as previously described (Allanki et al., 2021), slides were defrosted for 15 min at room temperature, washed twice with PBST, incubated in Bouin's solution for 2 h at 60°C and then stained according to the manufacturer's instructions (AFOG staining kit, BioGnost).

Primary antibodies used in this study include anti-Mef2 (rabbit, DZ01398, Boster Bio, 1:100), anti-PCNA (mouse, sc-56, Santa Cruz Biotechnology, 1:200), anti-RFP (rabbit, 600-401-379, Rockland, 1:200), N2.261 (mouse, 1:20, developed by H. M. Blau, obtained from the Developmental Studies Hybridoma Bank), anti-GFP (chicken, GFP-1010, Aves Labs, 1:500), anti-α-SMA (rabbit, GTX124505, GeneTex, 1:200), anti-FLI1 (rabbit, ab133485, Abcam, 1:100), anti-Aldh1a2 (mouse, when combined with rabbit anti-Fli1, anti-pERK, anti-α-SMA, or anti-Vim antibodies; sc-393204, Santa Cruz, 1:50), anti-Aldh1a2 (rabbit, all other experiments involving Aldh1a2 staining; GTX124302, GeneTex, 1:200), anti-pERK (rabbit, 4370, Cell Signaling Technology, 1:100), anti-Vim (rabbit, GTX133061, GeneTex, 1:150), anti-CAV1 (mouse, 610407, BD Transduction Laboratories 1:150) and anti-MHC (mouse, MF20, 14-6503-82, Invitrogen, 1:150). Alexa Fluor-conjugated secondary antibodies raised in goat (Thermo Fisher Scientific, 1:400; Table S5) and Phalloidin-Alexa 568-conjugated (A12380, Thermo Fisher Scientific, 1:200) were also used.

Fluorescence imaging of stained sections was performed using a Leica Thunder Imager, a Zeiss Cell Observer Spinning Disk inverted confocal microscope, a Nikon Ni-E ECLIPSE widefield microscope equipped with a SlideExpress 2 slideloader (Märzhäuser), a SOLA light engine (Lumencor) and a DS-Qi2 Mono Digital Microscope camera (Nikon). AFOG-stained sections were imaged using a Nikon SMZ25 microscope coupled with a Nikon Digital Sight DS-Ri1 camera. Whole-mount imaging of juvenile hearts was performed using a Zeiss Lightsheet Z.1 and whole-mount imaging of adult hearts was performed using a Nikon SMZ25 coupled with a Nikon Digital Sight DS-Ri1 camera.

Quantifications were conducted in two or three non-consecutive sections per ventricle with the ZEN Blue software and Fiji (ImageJ) after randomization of image files using Fiji plugins for blinded analysis. For the analysis of cEC proliferation, the percentage of proliferating cECs was determined by calculating the ratio of proliferating cECs to the total number of cECs in the BZI (200 µm of the border zone plus injured area). For CM dedifferentiation and proliferation analysis, the percentage of proliferating or dedifferentiating CMs was calculated as the ratio of proliferating or dedifferentiating CMs to the total number of CMs in 100 µm of the border zone. For analyses relating to endocardial expansion, the area of Aldh1a2^+^ or pERK^+^ endocardium was determined by applying a threshold using Fiji to exclusively select the fluorescent area, which was then divided by the total injured area. The cortical signal from the epicardium was excluded. The area occupied by Aldh1a2/Fli1 double-positive cells (excluding cortical endothelial cells) within the injured area was outlined, measured and then divided by the total injured area. For EdC proliferation analysis, the total number of Fli1/PCNA double-positive cells within the injured area (excluding cortical endothelial cells) was counted and then divided by the total injured area. For the analysis of pERK^+^ coronaries, the number of pERK^+^ coronary-like structures within 200 µm of the border zone and 200 µm inside the injured area was counted, and then divided by the total number of coronaries within the total 400 µm range. For the percentage of *egr3>EGFP* expressing EdCs, the number of EGFP/Aldh1a2 double-positive cells (excluding superficial cells) within the injured tissue was counted, and then divided by the total number of Aldh1a2^+^ cells. For general quantification of α-SMA^+^ cells, the number of intraventricular α-SMA^+^ cells (excluding superficial cells) within the injured tissue was counted and then divided by the total injured area. For quantification of endocardial-associated α-SMA^+^ cells, the number of α-SMA^+^ cells (excluding superficial cells) colocalized with, in morphological continuity with or in close proximity to Aldh1a2^+^ cells within the injured area was counted then divided by the total injured area. The number and length of CM protrusions were counted and measured starting from the injury border and extending towards the injured tissue, as previously described (Beisaw et al., 2020). For scar area analyses, the average ratio of the two or three scars with the biggest areas to the total ventricular area was calculated using Fiji. Scar areas were categorized into groups that were ranked based on their size. Scar composition analysis was performed as previously described (Koth et al., 2020), color images of AFOG-stained sections were processed using Fiji and split up into red, green and blue channels, and then thresholded using the same settings for all sections to exclusively select the areas of the respective colors and calculate the respective percentage of the total injured areas. For coronary coverage analysis and volume calculation of juvenile hearts, the 3D image files were processed using ZEN Blue and analyzed using Imaris 10.1, which allows the calculation of total coronary length encompassing both the bulbus arteriosus and the ventricle, as well as the measurement of ventricular volume. Coronary coverage was then determined by dividing the total vascular length by the ventricular volume. For coronary coverage analysis of untouched adult hearts, the area of coronaries covering both the ventral and dorsal sides of the ventricle was determined by applying a threshold on the projected whole-mount images using Fiji to exclusively select the coronary fluorescent area, which was then divided by the total area of both sides. For coronary coverage analysis of cryoinjured adult hearts, the area of coronaries covering the injured area was determined by applying a threshold on the projected whole-mount images using Fiji to exclusively select the coronary fluorescent area, which was then divided by the total injured area.

### Bulk RNA sequencing and analysis

*flt1^+/+^* and *flt1^−/−^* hearts were dissected at 96 hpci. The border zone and injured area were used for RNA sequencing. A pool of five ventricles was used per biological replicate. Total RNA was isolated using the miRNeasy micro Kit (Qiagen) combined with on-column DNase digestion (RNase-Free DNase Set, Qiagen) to avoid contamination by genomic DNA. RNA and library preparation integrity were verified with LabChip Gx Touch 24 (Perkin Elmer). 4 µg of total RNA was used as the input for VAHTS Stranded mRNA-seq V6 Library preparation following the manufacturer's protocol (Vazyme). Sequencing was performed on a NextSeq2000 instrument (Illumina) with 1×72 bp single end setup.

Trimmomatic version 0.39 was employed to trim reads after a quality drop below a mean of Q15 in a window of five nucleotides and keeping only filtered reads longer than 15 nucleotides (Bolger et al., 2014). Reads were aligned versus Ensembl zebrafish genome version danRer11 (Ensembl release 104) with STAR 2.7.10a (Dobin et al., 2013). Aligned reads were filtered to remove duplicates with Picard 3.0.0, multi-mapping, ribosomal or mitochondrial reads. Gene counts were established with featureCounts 2.0.4 by aggregating reads overlapping exons and excluding those overlapping multiple genes (Liao et al., 2014). The raw count matrix was normalized with DESeq2 version 1.36.0 (Love et al., 2014). Contrasts were created with DESeq2 based on the raw count matrix. All downstream analyses were based on the normalized gene count matrix. Volcano plots were produced to highlight DEG expression. A global clustering heatmap of samples was created based on the euclidean distance of regularized log transformed gene counts. Differentially expressed genes (adjusted *P* value<0.05) were selected for transcriptomic analysis (see Table S1). Gene Ontology and KEGG pathway analyses were performed using the Database for Annotation, Visualization and Integrated Discovery (DAVID). The RNA-Seq data generated in this study have been deposited in the GEO database under accession number GSE264406.

### Real-time quantitative polymerase chain reaction (RT-qPCR)

For RT-qPCR for validating the expression of genes of interest from RNA sequencing results, RNA was isolated from three pooled BZI tissues per biological replicate; for RT-qPCR for validating *egr3* expression in *egr3*-OE and *Tg(hsp70l:Cre);egr3^flox/+^*, RNA was isolated from one ventricle per biological replicate. RNA clean and concentrator extraction kit (Zymo) was used for RNA extraction. 250 ng of RNA was reverse transcribed using a Maxima First Strand cDNA Synthesis Kit (Thermo Fisher Scientific) following the manufacturer's protocol. RT-qPCR reactions were performed using the DyNAmo Flash SYBR Green qPCR master mix (Thermo Fischer Scientific) on a CFX Connect Real-Time BioRad machine. mRNA levels were normalized against the housekeeping gene *rpl13*. Primer sequences are listed in Table S2. Average Ct values of all RT-qPCR data are listed in Table S3.

### Single-cell RNA sequencing dataset analysis

The re-analysis of a published single-cell RNA sequencing dataset (GSE138181) (Koth et al., 2020) was carried out using the ADATA object provided by the Koth et al. and raw matrices were downloaded from GEO. The raw matrices were mapped to the normalized data from Koth et al. Compatibility of the raw matrix with the normalized subset was checked before adding the raw matrix to the ADATA to ensure that the order of genes and cells matched, and that the resulting dimensions of the subset matched the reference matrix. Mean expression of the cohorts was calculated using the normalized X-values from the provided ADATA file. *egr3* mean expression values per subset was calculated. Matrix plots and UMAPs were generated using Scanpy [scanpy.pl.matrixplot(), sc.pl.embedding()]. Violin plots were generated using Seaborn.

### Quantification and statistical analysis

All statistical analyses were performed using GraphPad Prism (v.10). Distribution of data in each group was assessed using the Shapiro-Wilk normality test. Unpaired, two-tailed Student's *t*-tests were applied for comparing two samples with normally distributed data; a Mann–Whitney *U* test was applied for comparing two samples with data not normally distributed. One-way ANOVA and Tukey's post-hoc tests were applied to multiple comparisons. Significance level was set to 0.05 for all tests. The exact *P* values are indicated in the figures. Data are mean±s.e.m. or mean±s.d., as indicated in the legends.

## Supplementary Material



10.1242/develop.203028_sup1 Supplementary information

Table S1. List of genes identified as significantly regulated (*padj* < 0.05) in RNA sequencing data.

Table S4. Proportion of EGFP^+^ EdCs within the wound of cryoinjured *egr3*>*EGFP* ventricles on each section (related to Figure 4).

Table S5. List of reagents and resources.
