# Supplementary material for: flt1 inactivation promotes zebrafish cardiac regeneration by enhancing endothelial activity and limiting the fibrotic response
Source: Development. 2024 Nov 29;151(23):dev203028. doi: 10.1242/dev.203028 (PMC11634031; doi:10.1242/dev.203028)
Supplement: Supplementary information [file develop-151-203028-s1.pdf]

Figure S1

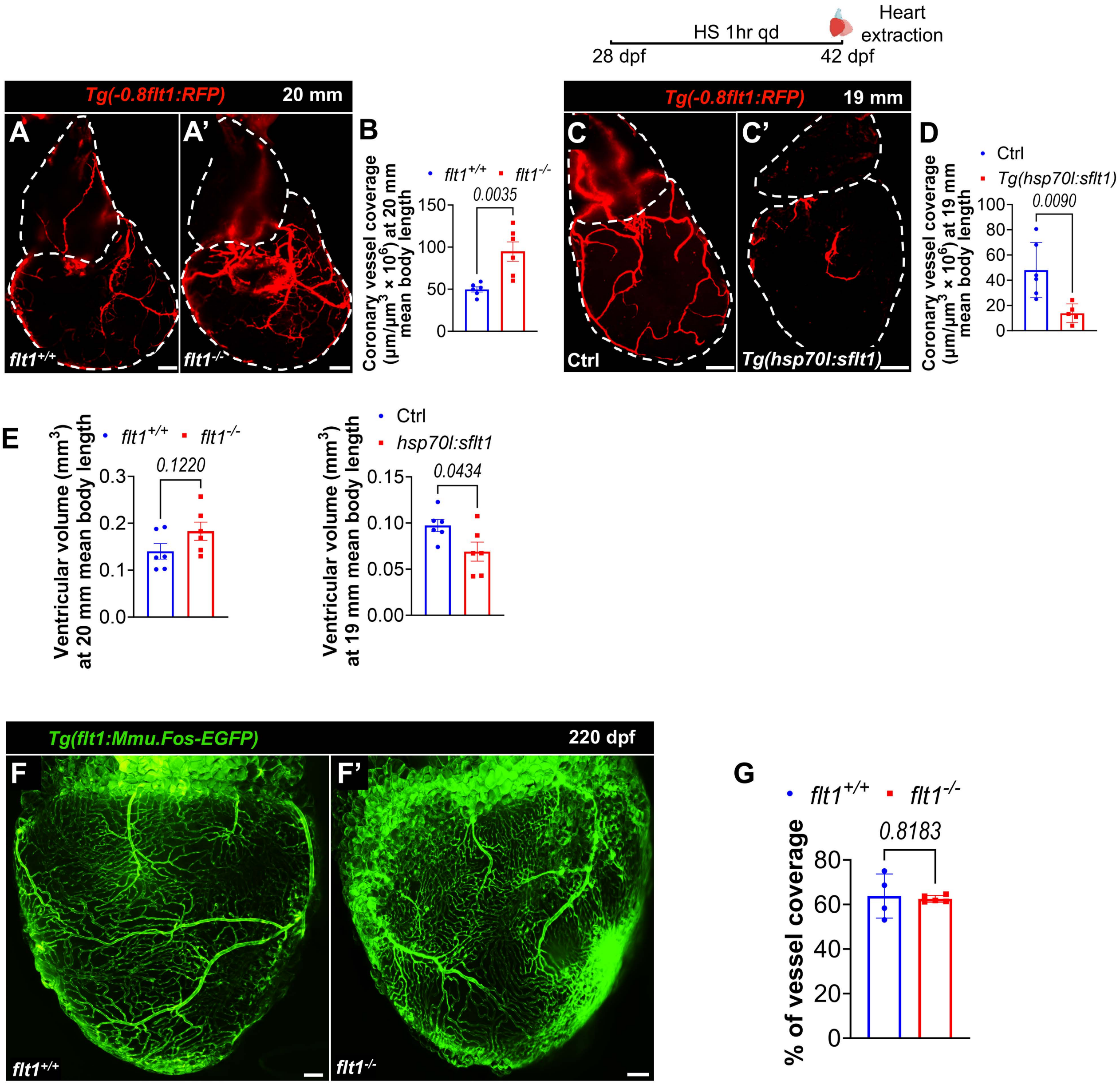

Development • Supplementary information

Fig. S1. Flt1 negatively regulates coronary formation during development

(A,A',C,C') Wholemount images of hearts from sibling *Tg(-0.8flt1:RFP);flt1*$^{+/+}$ (n=6, A) and *Tg(-0.8flt1:RFP);flt1*$^{-/-}$ (n=6, A') zebrafish, and from *Tg(-0.8flt1:RFP)* (Ctrl, n=6, C) and *Tg(-0.8flt1:RFP);Tg(hsp70l:sflt1)* (n=5, C') zebrafish at 42 dpf (body length: 19-20 mm). To ensure a sustained *sflt1* overexpression during the initiation of coronary network formation, we implemented a daily heat shock treatment from 28 to 42 dpf. **(B,D)** Quantification of coronary vessel coverage (ventricle and bulbus arteriosus) in the indicated genotypes at 42 dpf. **(E)** Quantification of ventricular volume in the heart of the indicated genotypes at 42 dpf. **(F,F')** Wholemount images of ventricles from sibling *Tg(flt1:Mmu.Fos-EGFP);flt1*$^{+/+}$ (n=4, F) and *Tg(flt1:Mmu.Fos-EGFP);flt1*$^{-/-}$ (n=5, F') adult zebrafish. **(G)** Quantification of coronary vessel coverage of adult ventricles from the indicated genotypes. White dotted lines in (A,A',C,C') outline the bulbi arteriosi and ventricles. HS, heat shock; qd, per day. Scale bars: 100 µm. Data show mean ± SEM (two-tailed, unpaired Student's *t*-test with *p* values shown in the graphs).

Development • Supplementary information

Figure S2

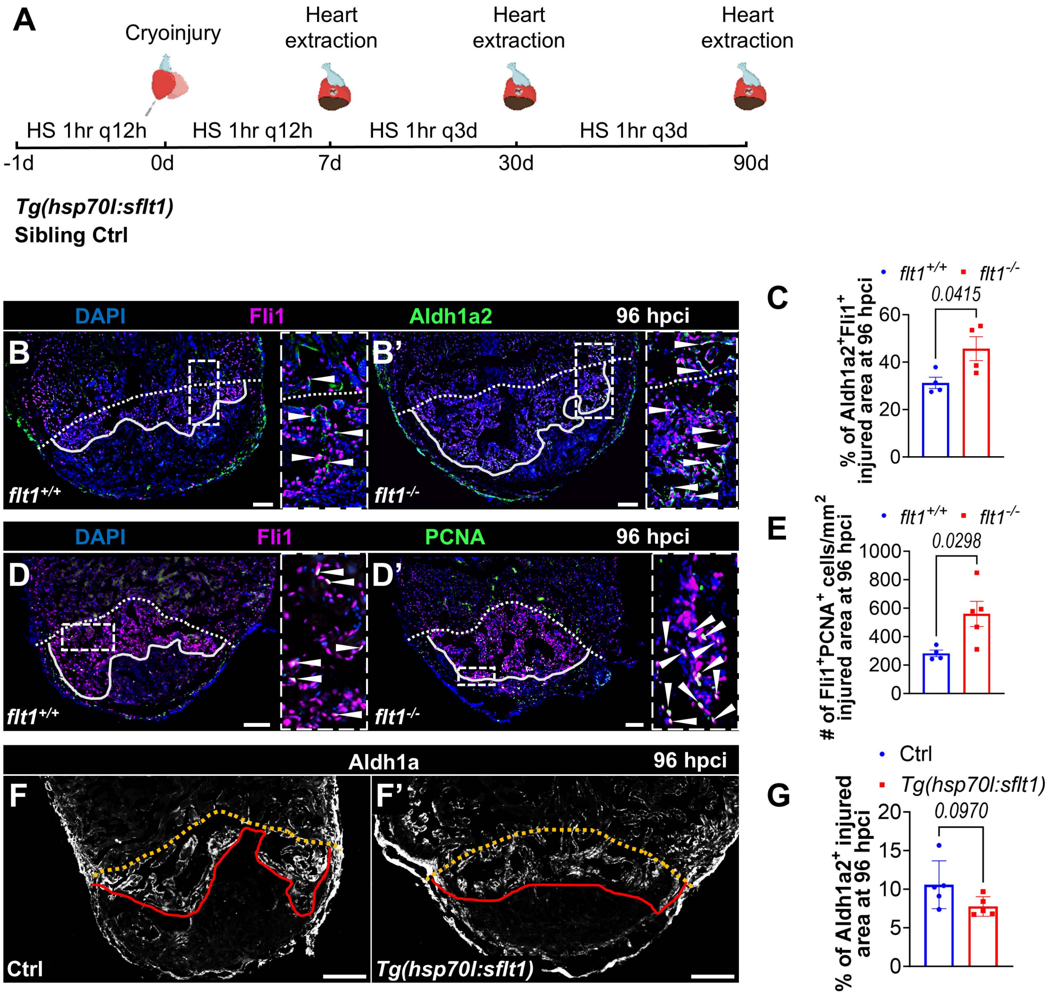

Development • Supplementary information

Fig. S2. Flt1 regulates endocardial expansion after cardiac cryoinjury

(A) Schematic diagram showing the heat shock treatment and cardiac cryoinjury on *Tg(hsp70l:sflt1)* zebrafish and control siblings (generated with BioRender.com). **(B,B')** Immunostaining of cryoinjured ventricles from *flt1*$^{+/+}$ (n=4, B) and *flt1*$^{-/-}$ (n=4, B') zebrafish at 96 hpci for Fli1 (cEC/EdC nuclei, magenta) and Aldh1a2 (activated EdCs, green) with DAPI (blue) counterstaining. White solid lines demarcate the Aldh1a2^{+}Fli1^{+} injured area. Arrowheads point to Aldh1a2^{+} EdCs. **(C)** Percentage of Aldh1a2^{+}Fli1^{+} injured area in ventricle sections from the indicated genotypes at 96 hpci. **(D,D')** Immunostainings of cryoinjured ventricles from *flt1*$^{+/+}$ (n=4, D) and *flt1*$^{-/-}$ (n=5, D') zebrafish at 96 hpci for Fli1 (cEC/EdC nuclei, magenta), PCNA (proliferation marker, green) with DAPI (blue) counterstaining. White solid lines demarcate the Fli1^{+} injured area. Arrowheads point to PCNA^{+} EdCs. **(E)** Quantification of Fli1^{+}PCNA^{+} cell number within the injured area on ventricle sections from the indicated genotypes. **(F,F')** Immunostaining for Aldh1a2 (activated endocardium, white) on sections of cryoinjured ventricles from non-transgenic Ctrl (n=5, F) and *Tg(hsp70l:sflt1)* (n=5, F') at 96 dpci. Red lines demarcate the extent of activated endocardium in the injured area. **(G)** Percentage of Aldh1a2^{+} injured area from the indicated genotypes. White and Orange dotted lines demarcate the injured areas. White dotted boxes correspond to the magnified regions. HS, heat shock; q12h, every 12 hours; q3d, every 3 days. Scale bars: 100 µm. Data show mean ± SEM (two-tailed, unpaired Student's *t*-test with *p* values shown in the graphs).

Development • Supplementary information

Figure S3

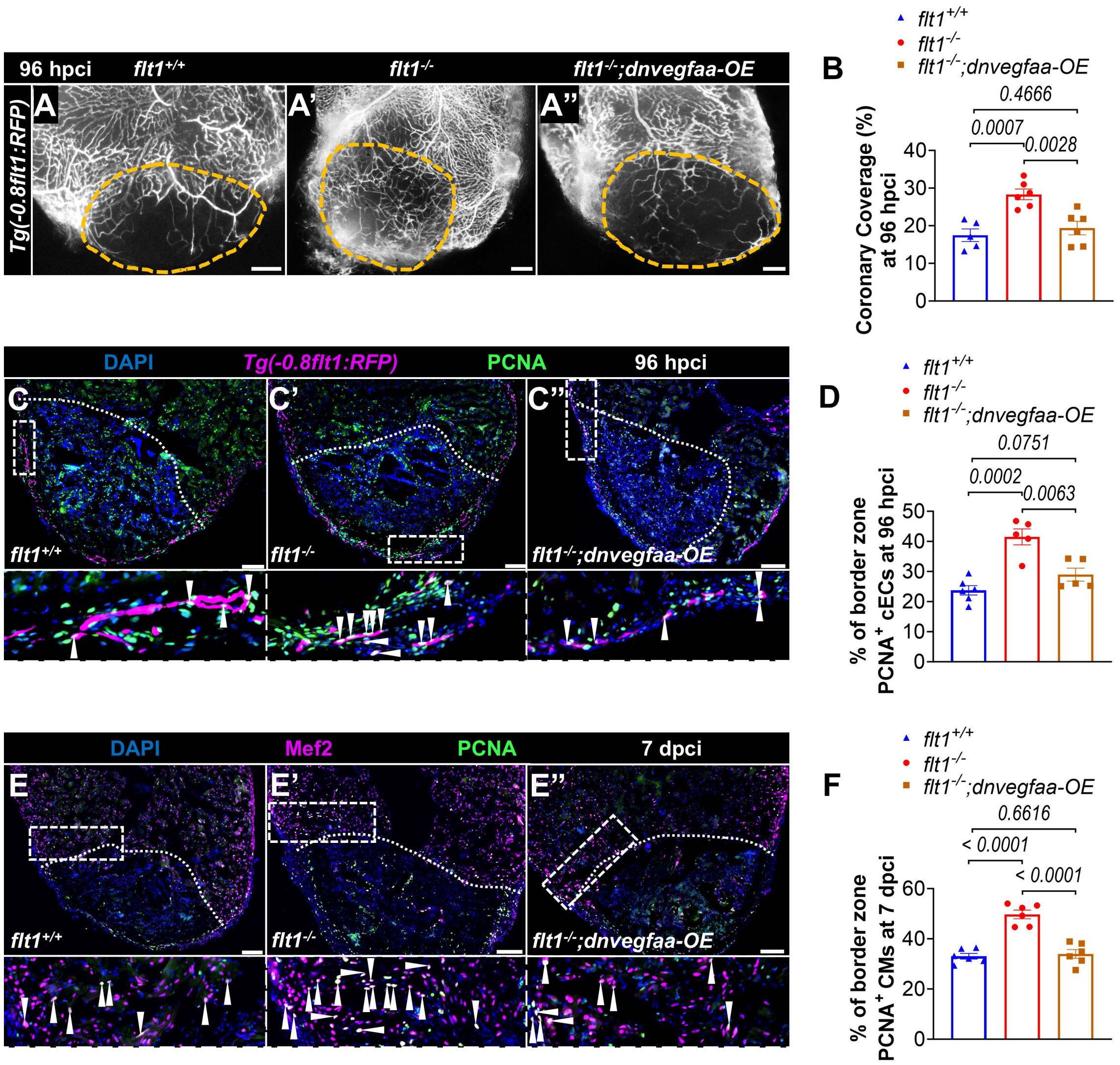

Development • Supplementary information

Fig. S3. Blockade of Vegfa signaling reverses the *flt1* mutant phenotypes

(A-A") Wholemount images of cryoinjured *Tg(-0.8flt1:RFP);flt1*$^{+/+}$ (n=5, A), *Tg(-0.8flt1:RFP);flt1*$^{-/-}$ (n=6, A'), and *Tg(-0.8flt1:RFP);Tg(hsp70l:dnvegfaa);flt1*$^{-/-}$ (n=6, A") ventricles showing revascularization of the injured area at 96 hpci. **(B)** Percentage of coronary vessel coverage of the wound in cryoinjured ventricles from the indicated genotypes at 96 hpci. **(C-C")** Immunostaining of cryoinjured ventricles from *Tg(-0.8flt1:RFP);flt1*$^{+/+}$ (n=6, C), *Tg(-0.8flt1:RFP);flt1*$^{-/-}$ (n=5, C'), and *Tg(-0.8flt1:RFP); Tg(hsp70l:dnvegfaa);flt1*$^{-/-}$ (n=5, C") zebrafish at 96 hpci for RFP (cECs, magenta), PCNA (proliferation marker, green) with DAPI (blue) counterstaining. Arrowheads point to PCNA^{+} cECs. **(D)** Percentage of PCNA^{+} cECs in the BZI of ventricle sections from the indicated genotypes at 96 hpci. **(E-E")** Immunostaining of cryoinjured ventricles from *flt1*$^{+/+}$ (n=6, E), *flt1*$^{-/-}$ (n=6, E'), and *Tg(hsp70l:dnvegfaa);flt1*$^{-/-}$ (n=6, E") zebrafish at 7 dpci for Mef2 (CM nuclei, magenta), PCNA (proliferation marker, green) with DAPI (blue) counterstaining. Arrowheads point to PCNA^{+} CMs. **(F)** Percentage of PCNA^{+} CMs in the border zone of ventricle sections from the indicated genotypes at 96 hpci. Orange (A-A") and white (C-C", E-E") dotted lines demarcate the injured areas. White dotted boxes in (C-C", E- E") correspond to the magnified regions. Scale bars: 100 µm. Data show mean ± SEM (one-way ANOVA and Tukey's post hoc tests with *p* values shown in the graphs).

Development • Supplementary information

Figure S4

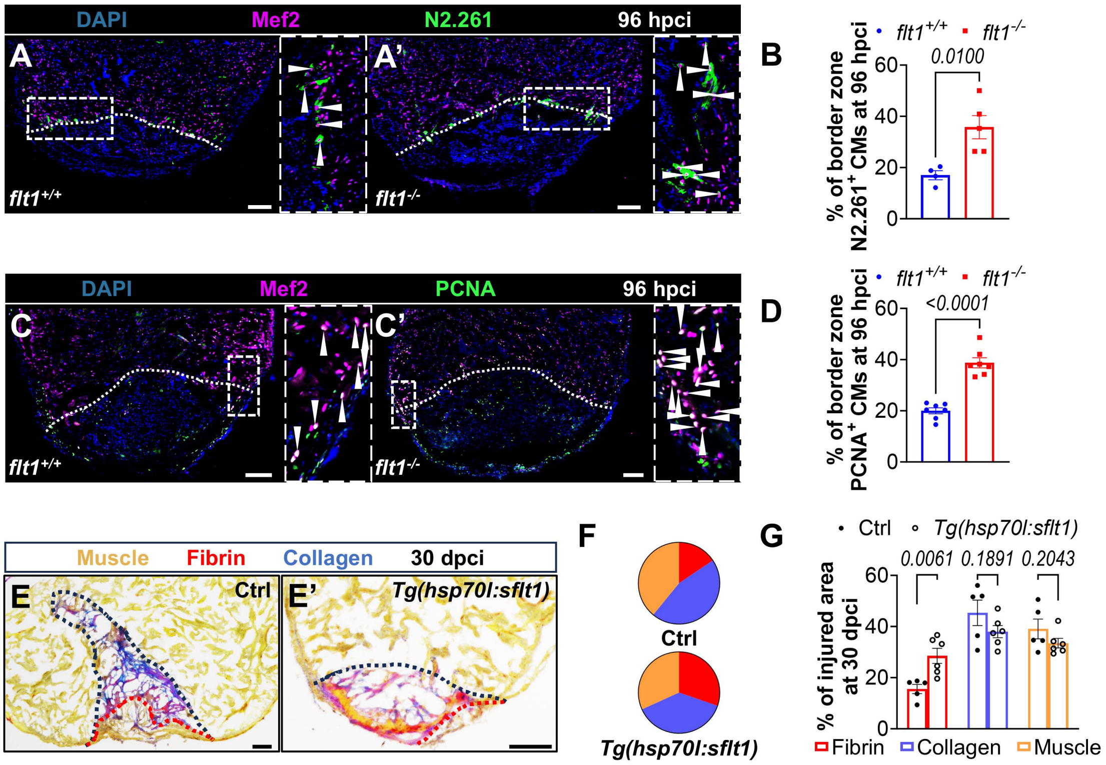

Fig. S4. ***flt1* modulation alters cardiomyocyte regeneration and scarring after cardiac cryoinjury**

(A,A') Immunostaining for Mef2 (CM nuclei, magenta) and N2.261 (embryonic myosin heavy chain, green) with DAPI (blue) counterstaining on sections of cryoinjured *flt1*$^{+/+}$ (n=4, A) and *flt1*$^{-/-}$ (n=5, A') ventricles at 96 hpci. Arrowheads points to N2.261^{+} CMs. **(B)** Percentage of N2.261^{+} CMs in the border zone of ventricle sections from the indicated genotypes at 96 hpci. **(C,C')** Immunostaining for Mef2 (CM nuclei, magenta) and PCNA (proliferation marker, green) with DAPI (blue) counterstaining on sections of cryoinjured *flt1*$^{+/+}$ (n=7, C) and *flt1*$^{-/-}$ (n=7, C') ventricles at 96 hpci. Arrowheads points to PCNA^{+} CMs. **(D)** Percentage of PCNA^{+} CMs in the border zone of ventricle sections from the indicated genotypes at 96 hpci. **(E,E')** AFOG staining of ventricle sections from non-transgenic Ctrl (n=5, E) and *Tg(hsp70l:sflt1)* (n=6, E') sibling zebrafish at 30 dpci. Orange, muscle; red, fibrin; blue, collagen. Black and red dotted lines delineate scar and regenerated muscle wall areas, respectively. **(F,G)** Quantification of differences in wound composition of ventricle sections from the indicated genotypes at 30 dpci displayed in pie charts (F) and scatter graph (G). White dotted lines demarcate the injured areas. White dotted boxes in (A,A',C,C') correspond to the magnified regions. Scale bars: 100 µm. Data show mean ± SEM (two-tailed, unpaired Student's *t*-test with *p* values shown in the graphs).

Development • Supplementary information

Figure S5

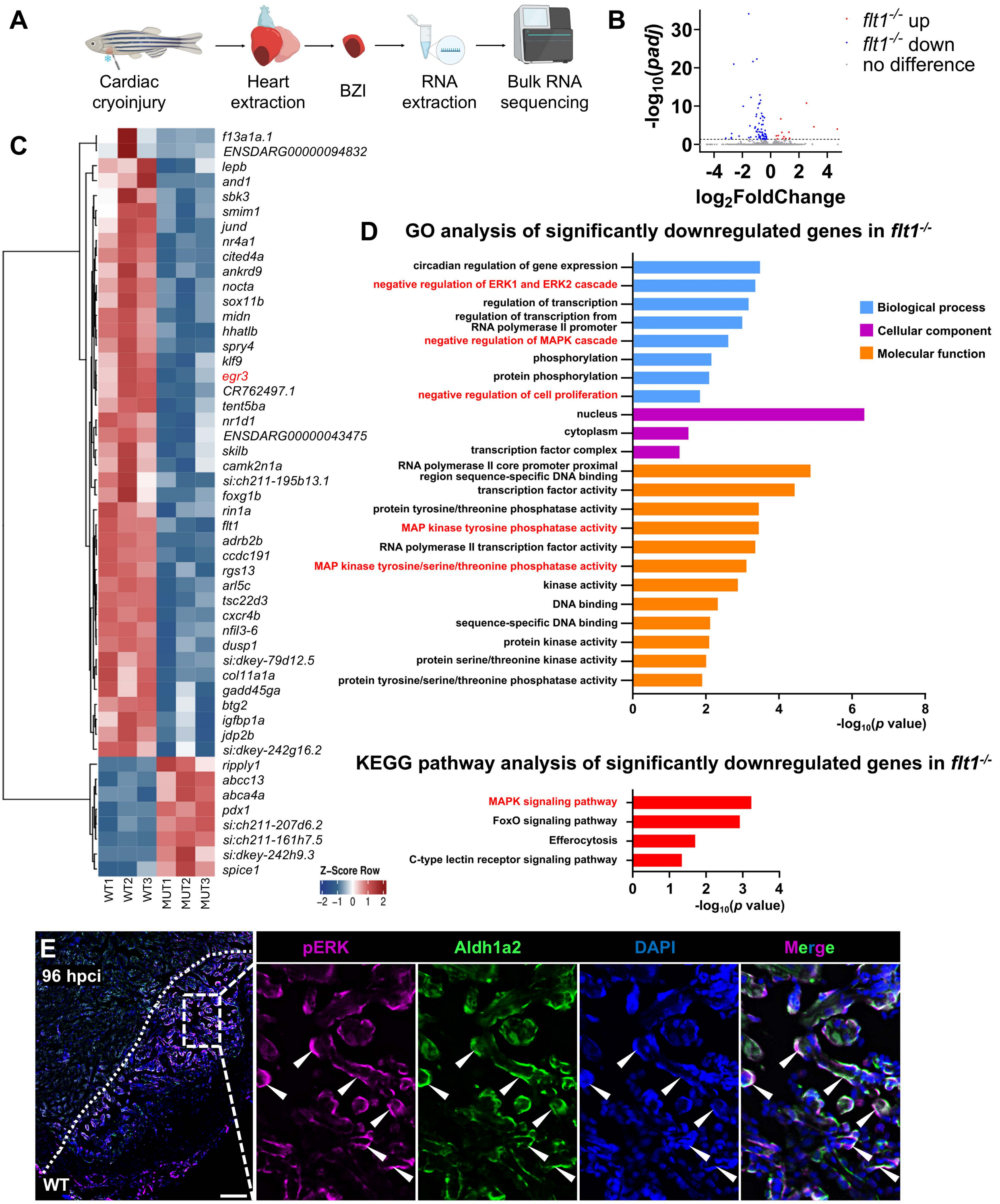

Development • Supplementary information

Fig. S5. *flt1* deletion promotes endothelial MAPK/ERK signaling and leads to the downregulation of anti-proliferative factors during cardiac regeneration

(A) Schematic diagram of experimental design for bulk RNA sequencing of the BZI of cryoinjured ventricles from sibling *flt1*$^{+/+}$ vs *flt1*$^{-/-}$ at 96 hpci (created with BioRender.com). **(B)** Volcano plot showing differentially expressed genes (adjusted $p \leq 0.05$) in *flt1*$^{-/-}$ compared with *flt1*$^{+/+}$. **(C)** Heat map showing top differentially expressed genes in injured hearts from sibling *flt1*$^{+/+}$ vs *flt1*$^{-/-}$ at 96 hpci. **(D)** Gene Ontology (GO) and KEGG pathway analysis of significantly downregulated genes (*padj* < 0.05) in *flt1*$^{-/-}$. **(E)** Immunostaining of a representative cryosectioned ventricle for pERK (phosphorylated ERK, magenta) and Aldh1a2 (activated EdC, green) with DAPI (blue) counterstaining at 96 hpci. Arrowheads point to the co-localization of pERK and Aldh1a2. White dotted lines demarcate the injured areas. White dotted boxes correspond to the magnified regions. *padj*, adjusted *p* value. Scale bars: 100 μm.

Development • Supplementary information

Figure S6

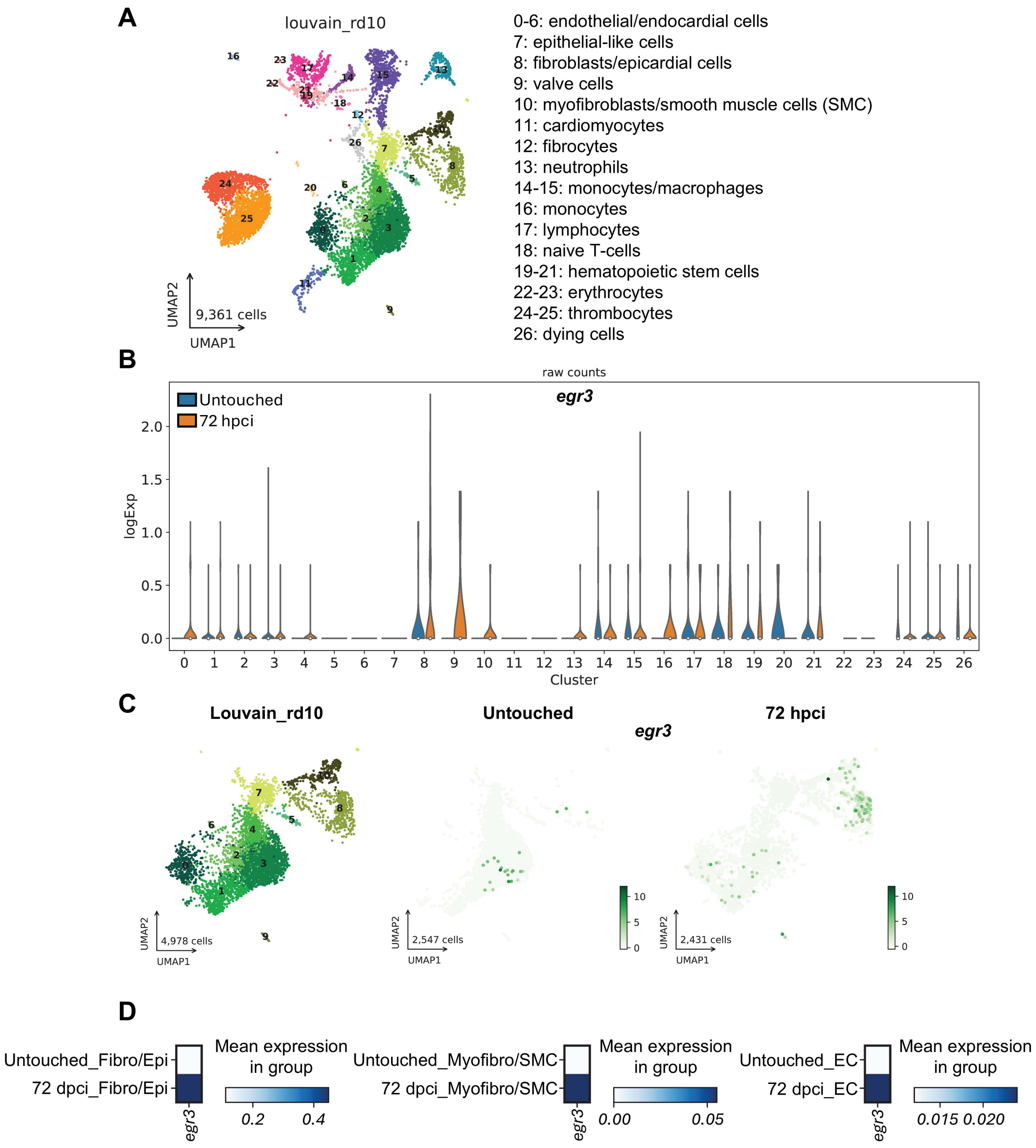

Fig. S6. *egr3* expression is induced after cardiac cryoinjury

(A,B) Analysis of the published single-cell RNA sequencing dataset (GSE138181) (Koth et al., 2020), showing the UMAP of all cells combined (A), and changes in *egr3* relative expression levels in all cell clusters in samples of untouched and cryoinjured ventricles at 72 hpci (B). **(C)** *egr3* expression in untouched and cryoinjured (72 dpci) ventricles in subcluster 1-10 highlighting *egr3* expression changes in endothelial clusters and fibroblast/myofibroblast clusters. **(D)** Matrix plots showing *egr3* expression changes in fibroblasts, myofibroblasts/SMCs, and endothelial cells (EC).

Development • Supplementary information

Figure S7

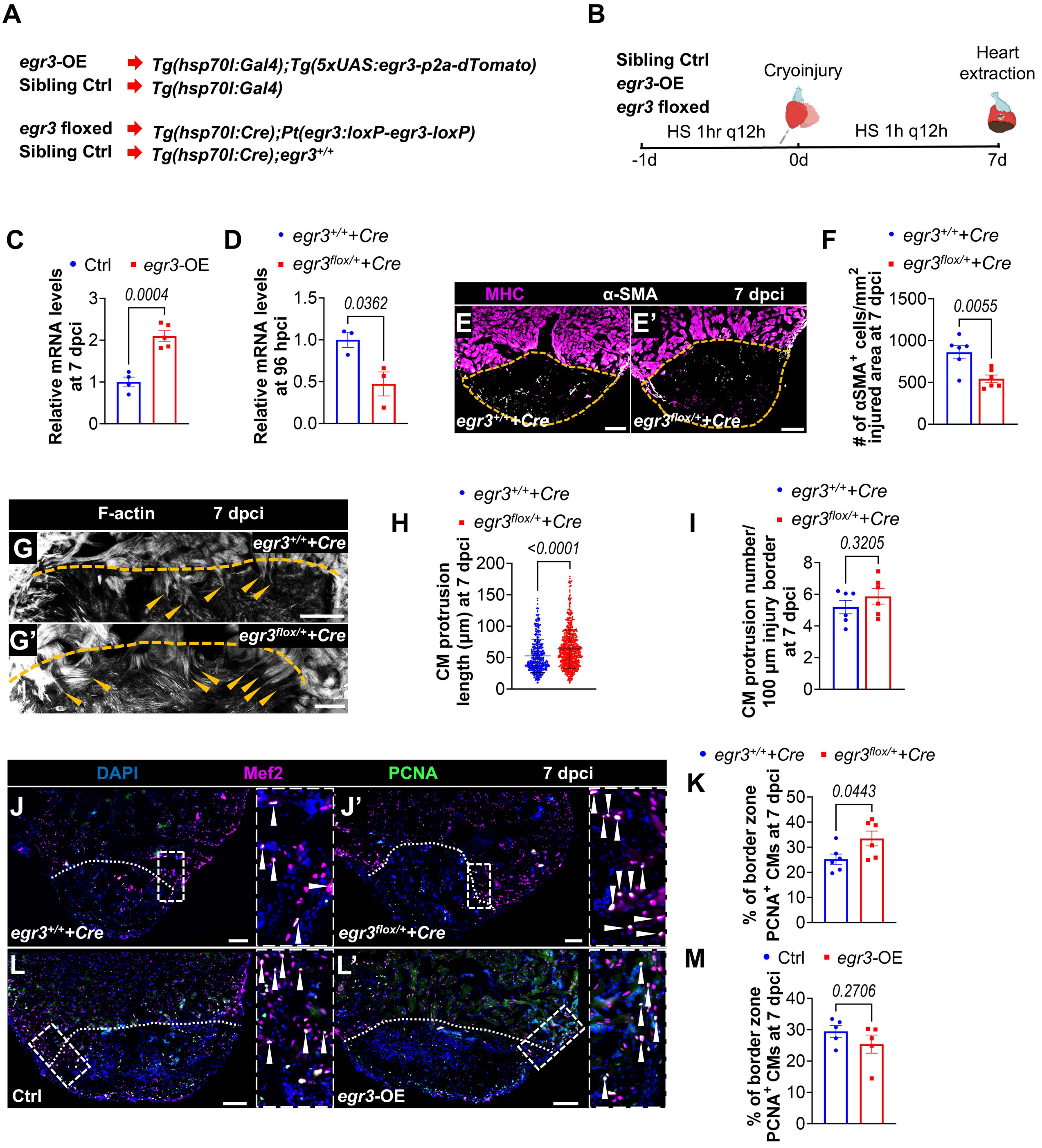

Development • Supplementary information

Fig. S7. ***egr3*** **promotes myofibroblast differentiation and negatively modulates cardiomyocyte repopulation**

(A) Schematic diagram showing the *egr3*-OE and *egr3* floxed zebrafish, and their respective control siblings. **(B)** Schematic diagram of heat shock (HS) treatment and cardiac cryoinjury on *egr3*-OE and *egr3* floxed zebrafish, and their respective control siblings (generated with BioRender.com). **(C)** RT-qPCR validation of *egr3* expression level in *egr3*-OE zebrafish after heat shock treatment. **(D)** RT-qPCR validation of *egr3* expression level in *Tg(hsp70l:Cre);egr3*$^{flox/+}$ zebrafish ventricles after heat shock treatment. **(E,E')** Immunostaining for MHC (magenta) and α-SMA (white) on sections of cryoinjured ventricles from *Tg(hsp70l:cre);egr3*$^{+/+}$ (n=6, E) and *Tg(hsp70l:Cre);egr3*$^{flox/+}$ (n=6, E') sibling zebrafish at 7 dpci. **(F)** Quantification of α-SMA^{+} cell number within the wound of the indicated genotypes at 7 dpci. **(G,G')** Phalloidin staining for F-actin (white) on 50-µm thick sections of cryoinjured ventricles from *Tg(hsp70l:cre);egr3*$^{+/+}$ (n=6, G) and *Tg(hsp70l:Cre);egr3*$^{flox/+}$ (n=6, G') sibling zebrafish at 7 dpci. Orange arrowheads point to the protruding CMs in the injured area. **(H,I)** Quantification of CM protrusion length (H) and number (I) in the indicated genotypes at 7 dpci. **(J,J',L,L')** Immunostaining for Mef2 (CM nuclei, magenta) and PCNA (proliferation marker, green) with DAPI (blue) counterstaining on sections of cryoinjured ventricles from *Tg(hsp70l:cre);egr3*$^{+/+}$ (n=6, J) and *Tg(hsp70l:Cre);egr3*$^{flox/+}$ (n=6, J') sibling zebrafish, and from *Tg(hsp70l:Gal4)* (Ctrl, n=5, L) and *egr3*-OE (n=5, L') sibling zebrafish at 7 dpci. **(K,M)** Percentage of PCNA^{+} CMs in the border zone of ventricle sections from the indicated genotypes at 7 dpci. Arrowheads point to PCNA^{+} CMs. Orange and white dotted lines demarcate the injured areas. White dotted boxes correspond to the magnified regions. Scale bars: 100 µm. Data in (C,D,I,K,M) show mean ± SEM (two-tailed, unpaired Student's *t*-test with *p* values shown in the graphs). Data in (H) show mean ± SD (two-tailed, Mann- Whitney *U* test with *p* values shown in the graphs).

Table S1. List of genes identified as significantly regulated (*padj* < 0.05) in RNA sequencing data.

Available for download at
https://journals.biologists.com/dev/article-lookup/doi/10.1242/dev.203028#supplementary-data

Development • Supplementary information

Table S2. RT-qPCR primers

Name	Sequence	Source
*gadd45ga*_qPCR_F	GGACAAGACAACACAGCTACTACTG	This paper
*gadd45ga*_qPCR_R	CCACGCTGTCTGGGTCAACATTC	This paper
*btg2*_qPCR_F	CTCAGAACACTACCAGCACCATTGG	This paper
*btg2*_qPCR_R	GTCCATTTCATGGTTGATCCGTATGC	This paper
*igfbp1a*_qPCR_F	AACCACAGCCAAAGCGAGACAGC	This paper
*igfbp1a*_qPCR_R	GGGACCCTGTTCCACCAGTTTC	This paper
*spry4*_qPCR_F	AGACGTCCGCTTTTACTCCG	This paper
*spry4*_qPCR_R	GCTCAGCATTTAGCACTCTTGC	This paper
*dusp1*_qPCR_F	CCCTCTGTATGATCAGGGTGGC	This paper
*dusp1*_qPCR_R	GTGGTCTTCAAAGTGGTTGGGACAG	This paper
*dusp4*_qPCR_F	CCATCGAATTCATAGACTCCGTCAAGG	This paper
*dusp4*_qPCR_R	CTCGAACTGCAGGAGTTGACCC	This paper
*dusp6*_qPCR_F	CTATCTCGAGGGTGGCTTCAGC	This paper
*dusp6*_qPCR_R	GGAGTCAGAGCTGATTCTGAGCC	This paper
*aplnra*_qPCR_F	CTCATTCCCGTGCTGTACATGCTC	This paper
*aplnra*_qPCR_R	GTAGCGGTCAAAACTCAGACAGGTC	This paper
*egr3*_qPCR_F	GAATACAGATAATATCATGGACTTGGGG	(da Silva et al., 2024)
*egr3*_qPCR_R	CTCACCAGGCTTATGATGTTGTTGTC	(da Silva et al., 2024)
*rpl13*_qPCR_F	TAAGGACGGAGTGAACAACCA	(El-Brolosy et al., 2019)
*rpl13*_qPCR_R	CTTACGTCTGCGGATCTTTCTG	(El-Brolosy et al., 2019)

Table S3. Average Ct values of RT-qPCRs

Fig. 3B		
	flt1$^{+/+}$	*flt1*$^{-/-}$
rpl13	20.03	19.58
btg2	23.34	25.19
igfbp1a	25.24	26.62
spry4	24.55	26.12
dusp1	23.26	24.89
rpl13	19.65	19.66
gadd45ga	24.14	26.30
rpl13	19.83	19.87
dusp4	26.08	26.75
rpl13	19.90	20.35
dusp6	22.57	23.72
aplnra	26.16	25.80
rpl13	19.40	19.81
egr3	28.84	30.31

Fig. S6C,D		
	Ctrl	*egr3*-OE
rpl13	17.86	17.11
egr3	29.08	27.24
	Ctrl	*egr3*$^{flox\text{-}rec/+}$
rpl13	21.18	20.64
egr3	32.55	33.27

Development • Supplementary information

Table S4. Proportion of EGFP$^+$ EdCs within the wound of cryoinjured *egr3>EGFP* ventricles on each section (related to Figure 4).

Available for download at
https://journals.biologists.com/dev/article-lookup/doi/10.1242/dev.203028#supplementary-data

Table S5. List of reagents and resources.

Available for download at
https://journals.biologists.com/dev/article-lookup/doi/10.1242/dev.203028#supplementary-data

References

da Silva, A. R., Gunawan, F., Boezio, G. L. M., Faure, E., Theron, A., Avierinos, J. F., Lim, S., Jha, S. G., Ramadass, R., Guenther, S., et al. (2024). egr3 is a mechanosensitive transcription factor gene required for cardiac valve morphogenesis. *Sci Adv* **10**, eadl0633.

El-Brolosy, M. A., Kontarakis, Z., Rossi, A., Kuenne, C., Gunther, S., Fukuda, N., Kikhi, K., Boezio, G. L. M., Takacs, C. M., Lai, S. L., et al. (2019). Genetic compensation triggered by mutant mRNA degradation. *Nature* **568**, 193-197.

Koth, J., Wang, X., Killen, A. C., Stockdale, W. T., Potts, H. G., Jefferson, A., Bonkhofer, F., Riley, P. R., Patient, R. K., Gottgens, B. and Mommersteeg, M. T. M. (2020). Runx1 promotes scar deposition and inhibits myocardial proliferation and survival during zebrafish heart regeneration. *Development* **147**.
