# Supplementary material for: flt1 inactivation promotes zebrafish cardiac regeneration by enhancing endothelial activity and limiting the fibrotic response
Source: Development. 2024 Nov 29;151(23):dev203028. doi: 10.1242/dev.203028 (PMC11634031; doi:10.1242/dev.203028)
Supplement: Supplementary file 1 [file develop-151-203028_review_history.pdf]

flt1 inactivation promotes zebrafish cardiac regeneration by enhancing endothelial activity and limiting the fibrotic response

Zhen-Yu Wang, Armaan Mehra, Qian-Chen Wang, Savita Gupta, Agatha Ribeiro da Silva, Thomas Juan, Stefan Günther, Mario Looso, Jan Detleffsen, Didier Y.R. Stainier and Rubén Marıń-Juez
DOI: 10.1242/dev.203028

Editor: Benoit Bruneau

Review timeline
Original submission: 8 May 2024
Editorial decision: 24 June 2024
First revision received: 18 October 2024
Accepted: 22 October 2024

Original submission

First decision letter

MS ID#: DEVELOP/2024/203028

MS TITLE: flt1 inactivation promotes zebrafish cardiac regeneration by enhancing endothelial activity and limiting the fibrotic response

AUTHORS: Zhen-Yu Wang, Armaan Mehra, Qian-Chen Wang, Agatha Ribeiro da Silva, Thomas Juan, Stefan Guenther, Mario Looso, Jan Detleffsen, Didier Y.R. Stainier, and Ruben Marin Juez

I have now received all the referees reports on the above manuscript, and have reached a decision. The referees' comments are appended below, or you can access them online: please go to BenchPress and click on the 'Manuscripts with Decisions' queue in the Author Area.

The overall evaluation is positive and we would like to publish a revised manuscript in Development, provided that the referees' comments can be satisfactorily addressed. Please attend to all of the reviewers' comments in your revised manuscript and detail them in your point-by-point response. All these revisions should be text or figure revisions only. If you do not agree with any of their criticisms or suggestions explain clearly why this is so.

Reviewer 1

Advance summary and potential significance to field

please see bellow my full report

Comments for the author

Wang et al. investigated the role of flt1, a decoy receptor for vascular endothelial growth factor A (VEGFA), in cardiac regeneration in zebrafish. Through genetic manipulation and cryoinjury experiments, the study demonstrated that deletion of flt1 enhances cardiac regeneration by promoting angiogenesis cardiomyocyte regeneration, and limiting scarring. Mechanistically, flt1 deletion led to enhanced Vegfa bioavailability and decreased expression of genes encoding

© 2024. Published by The Company of Biologists under the terms of the Creative Commons Attribution License (https://creativecommons.org/licenses/by/4.0/).

MAPK/ERK antagonists, thereby activating MAPK/ERK signaling in endothelial cells and downregulating the expression of the transcription factor egr3, which is associated with myofibroblast differentiation. The study highlights the importance of coronary and endocardial cells in facilitating tissue replenishment and underscores the interplay between endothelial cells, myofibroblasts, and cardiomyocytes in cardiac repair.

Overall the reviewer finds this paper suitable for publication in Development pending minor revisions. The most interesting aspect of this study is the part on egr3 acting downstream of VEGF signaling. The authors mention the use of recently developed gain- and loss-of-function genetic tools (Ribeiro da Silva et al., in press) to address the role of egr3 in cardiac regeneration. The results describing the effect of these lines on cardiac regeneration should be included in the current study.
It would be great if the authors could expand the intriguing part suggesting that egr3 can exert an inhibitory effect on cardiomyocyte proliferation, potentially by modulating myofibroblast-mediated ECM deposition. This would improve dramatically this paper.

The authors determine myofibroblast differentiation only using SMA staining.
Please add more markers of myofibroblast maturation, differentiation etc.

Minor points:
* The study compared flt1-/- with flt1+/+ and sflt1 overexpressing zebrafish. How do these findings align or differ from what is observed in mammals, particularly regarding scar formation and cardiomyocyte proliferation?
* To aid reader comprehension, please add a sentence introducing the membrane-bound form of flt1 compared to the soluble form in the Introduction section including the expression ratio of membrane-bound form versus soluble form in the context of cryoinjured zebrafish hearts.
* Please use the full term "endocardial cells" instead of the abbreviation "EdCs," as the entire manuscript does not mention what "EdCs" stands for.
* In an uninjured context, aside from affecting coronary vessel coverage (Fig. 1SA-D), does the perturbation of flt1 (using Tg(hsp70l:sflt1) and flt1 -/- models) impact other aspects of zebrafish heart function, such as heart morphology and cardiomyocyte proliferation?
* Could the authors discuss the long-term effects of enhanced cardiomyocyte proliferation in cryoinjured flt1-/- zebrafish? Are there any potential negative consequences?
* In line 156, it states, “However, while sflt1 overexpression strongly reduced cEC proliferation (Fig. 1G-H), it did not appear to affect endocardial expansion after cardiac cryoinjury (Fig. 1K-L),” which seems contradictory given the increased fibrotic area observed in sflt1 zebrafish at 90 dpci. The panels Fig. 1G-H examined endocardial expansion at 96 hpci. I wonder if the authors also examined endocardial expansion at a later time point (e.g., 7 dpci). If not please include this analysis to clarify the relationship between sflt1 overexpression, endocardial expansion, and fibrosis.
* In line 202, it states, "Gene Ontology and KEGG pathway analyses reveal the significant downregulation of genes associated with MAPK/ERK signaling," which gives readers the impression that the MAPK/ERK signaling pathway is likely inhibited in flt1-/- zebrafish. However, this is not the case. The pathways involved in the 'negative regulation of ERK1 and ERK2' and the 'negative regulation of the MAPK cascade' are downregulated in flt1-/- zebrafish (Fig. S4D), suggesting that the mechanisms responsible for inhibiting or dampening MAPK/ERK signaling are reduced in flt1-/- zebrafish compared to flt1+/+ zebrafish. Please revise this sentence.
* In Fig. 3E-G, the authors quantified the percentage of BZI Caveolin-1-positive and pERK positive cells within vessel-like structures. However, I am curious about how the authors defined vessel-like structures using the non-vessel-specific marker, Caveolin-1. It may be beneficial to utilize a more specific marker, such as CD31, to accurately label vessels.
* Please reduce the staining intensity of MHC in Fig. 3H-I (the inset panels) as it is currently challenging to discern the co-staining of MHC and EGFP.

Reviewer 2

Advance summary and potential significance to field

© 2024. Published by The Company of Biologists under the terms of the Creative Commons Attribution License (https://creativecommons.org/licenses/by/4.0/).

This is an excellent study that provides significant novel mechanistic insight into a highly interesting biological question. Using a variety of state-of-the art approaches the authors present an impressive number of very high-quality data that strongly support their conclusion that the VEGFR1 decoy receptor limits coronary vessel and endocardial cell regeneration in the zebrafish heart. They also provide evidence that a major target of VEGFR1 is the transcription factor egr3, and study its function using innovative tools (including a conditional, floxed allele, which are still very rarely used in zebrafish studies).

Comments for the author

I have two categories of issues that I would ask the authors to address:

1) a few that I think are essential to clarify a few ambiguous points and to exclude alternative explanations for observations 2) a “nice to have” suggestion, which would strengthen the paper, which however I don’t consider essential for acceptance considering the amount of work it represents and the already very high quality of the work

1) Major essential:

a) The authors show that the coronary vasculature develops differently (faster) in flt mutants. It is conceivable that this has rather major consequences for the interpretation of the regeneration phenotypes that they observe in the mutants. Maybe at least some of these are due to differences in anatomy of the hearts prior to injury and not due to specific functions of flt1 during regeneration? I thus consider it important that the authors provide data on the anatomy / morphology of adult noninjured flt mutant and control hearts specifically in respect to overall size, and distribution / density of the coronary vasculature.

b) Fig. 1C, D: how was the injured area identified in wild-type controls?

c) Fig. 1I-K & Fig.3C: results of measurements are heavily influenced by ability to unambiguously define the wound borders. How was this done? On the images presented it would not have been possible.

d) Fig. 3F & G: the same data are presented here twice, once in absolute numbers and second as %. It remains unclear a) how the latter was determined (% of what?) and b) why it would be informative to show both.

2) “Nice to have”

The authors propose that at least some of the beneficial effects of flt1 mutation are mediated by downregulation of egr3. This model could be strengthened by testing it using overexpression of egr3 in flt1 mutants.

3) Minor:

Line 250: spelling error: “they” instead of “there”

<u>Reviewer 3</u>

Advance summary and potential significance to field

In this manuscript, Wang and colleagues demonstrate that the inactivation of flt1, a receptor of VEGF, promotes cardiac regeneration in zebrafish by enhancing revascularization and limiting the fibrotic response. This study provides new insights into the importance of revascularization during heart regeneration and identifies new mechanisms involved in this process. Using complementary genetic models to manipulate flt1 expression, the authors show an enhanced proliferation of the coronary endothelium and rapid recovery of the coronary tree in the absence of flt1. In contrast, overexpression of a soluble form (sflt1) produces the opposite phenotype. A more detailed characterization of these animals demonstrates that deletion of flt1 (and thus, increased

© 2024. Published by The Company of Biologists under the terms of the Creative Commons Attribution License (https://creativecommons.org/licenses/by/4.0/).

availability of VEGF) results in enhanced cardiomyocyte proliferation and accelerated myocardial regeneration. Transcriptomic profiling identifies egr3 as one of the most downregulated genes in cryoinjured hearts from flt1 mutants compared to WT. The authors use a new knock-in line to identify the egr3+ cells which appear in a pattern compatible with cardiac fibroblasts. Overexpression of egr3 led to an increase in the number of myofibroblasts, while deletion of this gene reduced the frequency of these cells. The data suggests that in the absence of egr3 (mediated by flt1 deletion), the microenvironment becomes more favorable for cardiomyocyte proliferation by reducing myofibroblast differentiation from the endocardium.

Comments for the author

Overall, this is a beautifully written and very solid manuscript, and it may be the first time in many years as a reviewer that I don't have a long list of Comments for the authors. The text is written following a very nice flow of ideas. The approach and the methods are rigorous, and the presented data justify the conclusions. One of the main strengths is using multiple state-of-the-art genetic strains. If the authors want to put a cherry on the top, my only suggestion would be to document the phenotype of their egr3OE and egr3LOF models at ~30 or 60 days post cryoinjury using AFOG. Based on their data, one would hypothesize that egrOE animals would show more prominent scars rich in myofibroblasts. Conversely, the LOF model would probably exhibit the opposite phenotype. This would be an excellent end to this paper, if they have the time and the resources at hand.

Congratulations to the authors on a beautiful contribution to the field.

Juanma González-Rosa, PhD

First revision

<u>Author response to reviewers' comments</u>

Reviewer 1 Advance Summary and Potential Significance to Field:
please see bellow my full report

Reviewer 1 Comments for the Author:
Wang et al. investigated the role of flt1, a decoy receptor for vascular endothelial growth factor A (VEGFA), in cardiac regeneration in zebrafish. Through genetic manipulation and cryoinjury experiments, the study demonstrated that deletion of flt1 enhances cardiac regeneration by promoting angiogenesis, cardiomyocyte regeneration, and limiting scarring. Mechanistically, flt1 deletion led to enhanced Vegfa bioavailability and decreased expression of genes encoding MAPK/ERK antagonists, thereby activating MAPK/ERK signaling in endothelial cells, and downregulating the expression of the transcription factor egr3, which is associated with myofibroblast differentiation. The study highlights the importance of coronary and endocardial cells in facilitating tissue replenishment and underscores the interplay between endothelial cells, myofibroblasts, and cardiomyocytes in cardiac repair.

Overall the reviewer finds this paper suitable for publication in Development pending minor revisions. The most interesting aspect of this study is the part on egr3 acting downstream of VEGF signaling. The authors mention the use of recently developed gain- and loss-of-function genetic tools (Ribeiro da Silva et al., in press) to address the role of egr3 in cardiac regeneration. The results describing the effect of these lines on cardiac regeneration should be included in the current study.

We thank the reviewer for their supportive and constructive comments.

We have now included new data using *Pt(egr3:loxP-egr3-loxP)*^{bns661} homozygous animals. These new data confirm our previous results with *Pt(egr3:loxP-egr3-loxP)*^{bns661} hets and show that loss of *egr3* function induced in adult stages phenocopies the *flt1-/-* cardiac

© 2024. Published by The Company of Biologists under the terms of the Creative Commons Attribution License (https://creativecommons.org/licenses/by/4.0/).

regeneration phenotypes. Specifically, we observe a decreased number of aSMA^{+} cells (Fig. 4D-E) and increased CM protrusion length and proliferation (Fig. 5A-B,G-H). These data are now included in the manuscript.

It would be great if the authors could expand the intriguing part suggesting that egr3 can exert an inhibitory effect on cardiomyocyte proliferation, potentially by modulating myofibroblast- mediated ECM deposition. This would improve dramatically this paper.

We have now further elaborated on this point in the revised manuscript.

The authors determine myofibroblast differentiation only using SMA staining. Please add more markers of myofibroblast maturation, differentiation etc.

Thanks for the comment. We agree that having more resolution in this respect would be desirable. Unfortunately, currently the only marker for myofibroblasts used and validated in zebrafish is aSMA.

Minor points:
*The study compared flt1-/- with flt1+/+ and sflt1 overexpressing zebrafish. How do these findings align or differ from what is observed in mammals, particularly regarding scar formation and cardiomyocyte proliferation?

These points have now been mentioned in the revised version of the manuscript.

* To aid reader comprehension, please add a sentence introducing the membrane-bound form of flt1 compared to the soluble form in the Introduction section, including the expression ratio of membrane-bound form versus soluble form in the context of cryoinjured zebrafish hearts.

The introduction has now been modified to better introduce both *flt1* isoforms.

In terms of 'the expression ratio of membrane-bound form versus soluble form of *flt1* in the context of cryoinjured zebrafish hearts', we analyzed relevant RNAseq datasets (GSE94617) to quantify s*flt1* vs *flt1* (based on the exon that is unique to *sflt1* (which includes a 3'UTR)). Based on this analysis, it appears that *sflt1* is significantly more abundant than *flt1*, and that there *flt1/sflt1* mRNA levels appear to decrease after injury, but exact quantification is difficult. This difficulty is in part due to the bias towards the end of transcripts because of the use of polyA captured sequencing libraries. This interesting question will need to be addressed using another mRNA sequencing approach (eg, nanopore) and/or qPCR.

* Please use the full term "endocardial cells" instead of the abbreviation "EdCs," as the entire manuscript does not mention what "EdCs" stands for.

We have now clarified that EdCs refer to endocardial cells.

* In an uninjured context, aside from affecting coronary vessel coverage (Fig. 1SA-D), does the perturbation of flt1 (using Tg(hsp70l:sflt1) and flt1 -/- models) impact other aspects of zebrafish heart function, such as heart morphology and cardiomyocyte proliferation?

We have now quantified ventricular volume in uninjured hearts. *flt1* mutant ventricles displayed no significant difference when compared with wild-type siblings while *Tg(hsp70l:sflt1)*-expressing ventricles were significantly smaller (Fig. S1E). We observed no other obvious differences. These data have now been included in the revised version of the manuscript. We have also measured coronary coverage in untouched adult ventricles and found no significant difference between wild types and *flt1* mutant siblings (Fig. S1F-G).

* Could the authors discuss the long-term effects of enhanced cardiomyocyte proliferation in cryoinjured flt1-/- zebrafish? Are there any potential negative consequences?

Unfortunately, we did not analyse animals beyond 90 dpci. In untouched conditions, animals

© 2024. Published by The Company of Biologists under the terms of the Creative Commons Attribution License (https://creativecommons.org/licenses/by/4.0/).

are fertile and viable as reported by Matsuoka et al. 2016. (PMID: 27852438). While it will be interesting to look at later stages, we do not expect a continuous increase in cardiomyocyte proliferation.

* In line 156, it states, “However, while sflt1 overexpression strongly reduced cEC proliferation (Fig. 1G-H), it did not appear to affect endocardial expansion after cardiac cryoinjury (Fig. 1K-L),” which seems contradictory given the increased fibrotic area observed in sflt1 zebrafish at 90 dpci. The panels Fig. 1G-H examined endocardial expansion at 96 hpci. I wonder if the authors also examined endocardial expansion at a later time point (e.g., 7 dpci). If not, please include this analysis to clarify the relationship between sflt1 overexpression, endocardial expansion, and fibrosis.

Thanks for this suggestion. We have now performed these experiments and found that, indeed, endocardial expansion was reduced at 7 dpci in *sflt1* overexpression conditions when compared with control siblings. These new data have been included in the manuscript (Fig. 1 K-L and Fig. S2F-G) and mentioned in the text accordingly.

* In line 202, it states, "Gene Ontology and KEGG pathway analyses reveal the significant downregulation of genes associated with MAPK/ERK signaling," which gives readers the impression that the MAPK/ERK signaling pathway is likely inhibited in flt1-/- zebrafish. However, this is not the case. The pathways involved in the 'negative regulation of ERK1 and ERK2' and the 'negative regulation of the MAPK cascade' are downregulated in flt1-/- zebrafish (Fig. S4D), suggesting that the mechanisms responsible for inhibiting or dampening MAPK/ERK signaling are reduced in flt1-/- zebrafish compared to flt1+/+ zebrafish. Please revise this sentence.

Thanks for the suggestion. We have now corrected this sentence for clarity.

* In Fig. 3E-G, the authors quantified the percentage of BZI Caveolin-1-positive and pERK positive cells within vessel-like structures. However, I am curious about how the authors defined vessel-like structures using the non-vessel-specific marker, Caveolin-1. It may be beneficial to utilize a more specific marker, such as CD31, to accurately label vessels.

We have now repeated these analyses using zebrafish with the *flt1:Mmu.Fos-EGFP* transgene which is expressed specifically in the coronary endothelium. These new data have been included in the revised manuscript, replacing the previous Caveolin-1 based analyses (Fig. 3E-G).

* Please reduce the staining intensity of MHC in Fig. 3H-I (the inset panels) as it is currently challenging to discern the co-staining of MHC and EGFP.

We have now reduced the staining intensity of MHC to a point where all CMs are still visible. Further reducing it compromises the myocardial signal, especially in trabecular muscle. The EGFP and Aldh1a2 signals should be visible in the insets where the MHC signal has been excluded (Fig. 3I^{2,3}).

***** Reviewer 2 Advance Summary and Potential Significance to Field: This is an excellent study that provides significant novel mechanistic insight into a highly interesting biological question. Using a variety of state-of-the art approaches the authors present an impressive number of very high-quality data that strongly support their conclusion that the VEGFR1 decoy receptor limits coronary vessel and endocardial cell regeneration in the zebrafish heart. They also provide evidence that a major target of VEGFR1 is the transcription factor egr3, and study its function using innovative tools (including a conditional, floxed allele, which are still very rarely used in zebrafish studies).

Reviewer 2 Comments for the Author:
I have two categories of issues that I would ask the authors to address:

1) a few that I think are essential to clarify a few ambiguous points and to exclude alternative explanations for observations

© 2024. Published by The Company of Biologists under the terms of the Creative Commons Attribution License (https://creativecommons.org/licenses/by/4.0/).

2) a “nice to have” suggestion, which would strengthen the paper, which however I don’t consider essential for acceptance considering the amount of work it represents and the already very high quality of the work

We thank the reviewer for their supportive and constructive comments.

1) Major essential:

a) The authors show that the coronary vasculature develops differently (faster) in flt mutants. It is conceivable that this has rather major consequences for the interpretation of the regeneration phenotypes that they observe in the mutants. Maybe at least some of these are due to differences in anatomy of the hearts prior to injury and not due to specific functions of flt1 during regeneration? I thus consider it important that the authors provide data on the anatomy /morphology of adult noninjured flt mutant and control hearts, specifically in respect to overall size, and distribution / density of the coronary vasculature.

We did not observe obvious morphological changes in *flt1* mutant ventricles when compared with WT siblings. This point can also be observed in the different ventricular sections throughout the manuscript. We have now also measured ventricular volume in *flt1* mutants and found no significant difference when compared with WT siblings (Fig. S1E). Furthermore, we have now also quantified ventricular vessel coverage in untouched adult ventricles and found no significant difference between wild types and mutant siblings (Fig. S1F-G). All these data are now included in the revised version of the manuscript.

b) Fig. 1C, D: how was the injured area identified in wild-type controls?

The injured area can be identified based on the regenerated vascular morphology, which is distinct from the untouched in terms of density and pattterning (see Fig 1C and D), as well as by the tissue transparency at 30 dpci when the scar is still prominent. We have extensive experience performing these analyses (Marin-Juez et al. 2016 (PMID: 27647901), 2019 (PMID: 31743664), El-Sammak et al. 2022(PMID: 35264012)).

c) Fig. 1I-K & Fig.3C: results of measurements are heavily influenced by ability to unambiguously define the wound borders. How was this done? On the images presented it would not have been possible.

At early time points after cryoinjury, the injured area is easily identifiable by changes in tissue morphology. In addition, several types of staining allow for different ways to identify the border zone. In Fig. 1I-K and Fig. 3C, we stained for Aldh1a2 and pERK, respectively. After injury both the epicardium and especially the endocardium express these proteins, thereby allowing the easy identification of the injured area and border zone, as previously shown by others (e.g., Kikuchi et al. 2011 (PMID: 21397850), Cardeira- da-Silva et al. 2024 (PMID: 38684665)). To help the reader, we delineated the injured area in all panels, and relevant papers are referenced in the text.

d) Fig. 3F & G: the same data are presented here twice, once in absolute numbers and second as %. It remains unclear a) how the latter was determined (% of what?) and b) why it would be informative to show both.

Fig. 3G shows the percentage of borderzone pERK+ coronary endothelial cells (EGFP+), i.e., the proportion of coronary endothelial cells that express pERK over the total number of coronary endothelial cells after injury. Fig 3F shows the total number of borderzone pERK+ coronary endothelial cells (EGFP+). In both cases, we quantified 3 non- consecutive sections per ventricle and calculated the average value for each ventricle. This point is now clarified in the figure legend. These two quantifications are especially helpful for replication purposes since coronary revascularization can be variable.

2) “Nice to have”

© 2024. Published by The Company of Biologists under the terms of the Creative Commons Attribution License (https://creativecommons.org/licenses/by/4.0/).

The authors propose that at least some of the beneficial effects of flt1 mutation are mediated by downregulation of egr3. This model could be strengthened by testing it using overexpression of egr3 in flt1 mutants.

We agree that this would be a good experiment to do. Unfortunately, just getting the mutant animals with all the required transgenes ready for injuries would take a year.

3) Minor:

Line 250: spelling error: “they” instead of “there”
Noted and corrected. Thanks!

***** Reviewer 3 Advance Summary and Potential Significance to Field: In this manuscript, Wang and colleagues demonstrate that the inactivation of flt1, a receptor of VEGF, promotes cardiac regeneration in zebrafish by enhancing revascularization and limiting the fibrotic response.
This study provides new insights into the importance of revascularization during heart regeneration and identifies new mechanisms involved in this process.
Using complementary genetic models to manipulate flt1 expression, the authors show an enhanced proliferation of the coronary endothelium and rapid recovery of the coronary tree in the absence of flt1. In contrast, overexpression of a soluble form (sflt1) produces the opposite phenotype. A more detailed characterization of these animals demonstrates that deletion of flt1 (and thus, increased availability of VEGF) results in enhanced cardiomyocyte proliferation and accelerated myocardial regeneration. Transcriptomic profiling identifies egr3 as one of the most downregulated genes in cryoinjured hearts from flt1 mutants compared to WT. The authors use a new knock-in line to identify the egr3+ cells, which appear in a pattern compatible with cardiac fibroblasts. Overexpression of egr3 led to an increase in the number of myofibroblasts, while deletion of this gene reduced the frequency of these cells. The data suggests that in the absence of egr3 (mediated by flt1 deletion), the microenvironment becomes more favorable for cardiomyocyte proliferation by reducing myofibroblast differentiation from the endocardium.

Reviewer 3 Comments for the Author:
Overall, this is a beautifully written and very solid manuscript, and it may be the first time in many years as a reviewer that I don’t have a long list of comments for the authors. The text is written following a very nice flow of ideas. The approach and the methods are rigorous, and the presented data justify the conclusions. One of the main strengths is using multiple state-of-the-art genetic strains. If the authors want to put a cherry on the top, my only suggestion would be to document the phenotype of their egr3OE and egr3LOF models at ~30 or 60 days post cryoinjury using AFOG. Based on their data, one would hypothesize that egrOE animals would show more prominent scars rich in myofibroblasts. Conversely, the LOF model would probably exhibit the opposite phenotype. This would be an excellent end to this paper, if they have the time and the resources at hand.

Congratulations to the authors on a beautiful contribution to the field.

Juanma González-Rosa, PhD

We thank the reviewer for his encouraging words and suggestions.

We have now analyzed scarring in egr3-OE animals by AFOG staining at 90 dpci (Fig. 5I- K). As hypothesized by the reviewer, we found that these animals present a larger collagen scar compared with controls.

<u>Second decision letter</u>

MS ID#: DEVELOP/2024/203028

© 2024. Published by The Company of Biologists under the terms of the Creative Commons Attribution License (https://creativecommons.org/licenses/by/4.0/).

MS TITLE: flt1 inactivation promotes zebrafish cardiac regeneration by enhancing endothelial activity and limiting the fibrotic response

AUTHORS: Zhen-Yu Wang, Armaan Mehra, Qian-Chen Wang, Savita Gupta, Agatha Ribeiro da Silva, Thomas Juan, Stefan Guenther, Mario Looso, Jan Detleffsen, Didier Y.R. Stainier, and Ruben Marin Juez

I am happy to tell you that your manuscript has been accepted for publication in Development, pending our standard publication integrity checks.

© 2024. Published by The Company of Biologists under the terms of the Creative Commons Attribution License (https://creativecommons.org/licenses/by/4.0/).
